# Chromosome-level genome assembly of *Pontederia cordata* L. provides insights into its rapid adaptation and variation of flower colours

**DOI:** 10.1093/dnares/dsaf002

**Published:** 2025-01-29

**Authors:** Jia-Le Wang, Wen-Da Zhang, Xiao-Dong Yang, Pu-Guang Zhao, Xiang-Yu Wang, Shu-Ying Zhao, Ling-Yun Chen

**Affiliations:** School of Environment and Ecology, Jiangsu Open University, Nanjing 210036, China; School of Traditional Chinese Pharmacy, China Pharmaceutical University, Nanjing 211198, China; School of Traditional Chinese Pharmacy, China Pharmaceutical University, Nanjing 211198, China; School of Environment and Ecology, Jiangsu Open University, Nanjing 210036, China; School of Traditional Chinese Pharmacy, China Pharmaceutical University, Nanjing 211198, China; School of Traditional Chinese Pharmacy, China Pharmaceutical University, Nanjing 211198, China; School of Environment and Ecology, Jiangsu Open University, Nanjing 210036, China; School of Traditional Chinese Pharmacy, China Pharmaceutical University, Nanjing 211198, China

**Keywords:** *Pontederia cordata* L, chromosome-level genome, comparative genomic analysis, environmental adaptation, variation of flower colours

## Abstract

*Pontederia cordata* L. is an aquatic ornamental plant native to the Americas but has been widely distributed in South Asia, Australia, and Europe. The genetic mechanisms behind its rapid adaptation and spread have not yet been well understood. To understand the mechanisms for its rapid adaptation, this study assembled the first chromosome-level genome of *P. cordata*. The genome assembly, which spans 527.5 Mb, is anchored on 8 pseudochromosomes with a scaffold N50 of 48 Mb and encompasses 29,389 protein-coding genes. Further analyses revealed that *P. cordata* had experienced 3 whole-genome duplications (WGDs) events. These WGDs are associated with gene family expansion and increased numbers of resistance gene analogs and transcription factors. Positive selection analysis indicated that genes derived from tandem duplication (TD) and proximal duplication were more likely to undergo positive selection, and were enriched in plant defense and disease resistance. These results implied that WGDs, TD, and positive selection enhanced the environmental adaptability of *P*. *cordata*. In addition, we found that down-regulation of *F3ʹ5ʹH*, *DFR*, *ANS*, and *UFGT* likely caused the flower colour variation for *P. cordata* from violet to white. The first chromosome-level genome of *P. cordata* here provides a valuable genomic resource for investigating the rapid adaptation and flower colour variation of the species.

## 1. Introduction

*Pontederia cordata* L. is an aquatic ornamental plant in Pontederiaceae (Commelinales) and native to the American continent (Fig. S1).^1^ The plant has been introduced to many countries (e.g. China) or regions (e.g. Southeast Asia, Australia, and Europe) as a horticultural plant. It colonizes shallow waters and disperses through rapid vegetative propagation.^2^ In some areas where the plant is cultivated, it exhibits invasive behaviour.^3^

Exploring the genetic basis of plants is important for understanding the factors that contributed to their successful expansion into new habitats.^4^ Extensive research has demonstrated that expanding genetic material may result in key innovations and phenotypic novelties, which could enhance adaptation and survival.^5–8^ Positive selection, another driving force in adaptive evolution, occurs when advantageous gene mutations are retained and accumulated through natural selection.^9^ Numerous studies showed that genes undergoing positive selection are involved in processes directly relevant to environmental adaptation, such as stress resistance^10^ and disease resistance.^11^ Plants can perceive and respond to environmental changes and stresses through complex biological processes.^12^ Resistance gene analogs (RGAs) can confer disease resistance in plants and often form clusters and frequently change gene copy numbers among different species.^13–15^ Moreover, transcription factors (TFs) are also essential for plant adaptation, as they can regulate gene expression in response to environmental changes.^16^ To date, the adaptation mechanisms of several aquatic plants have been investigated (e.g. *Acorus gramineus*,^17^*Lemna minuta*,^18^*Wolffia australiana*,^19^ and *Pistia stratiotes*^20^). However, the mechanisms underlying the rapid adaptation of *P*. *cordata* remain largely unexplored.

Flower colour is a significant trait of ornamental plants. It is mainly determined by the types and levels of anthocyanins and their derivatives.^21^ Anthocyanins are a kind of flavonoid, and their biosynthetic pathway has been well studied.^22^ The upstream enzymes in the anthocyanins biosynthesis pathway include chalcone synthase (CHS), chalcone isomerase (CHI), and flavanone 3-hydroxylase (F3H). The downstream enzymes include flavonoid-3ʹ,5ʹ-hydroxylase (F3ʹ5ʹH), dihydroflavonol-4-reductase (DFR), anthocyanidin synthase (ANS), and flavonoid-3-O-glycosyltransferase (UFGT). In the floriculture industry, developing bluish-coloured flowers in desirable plants has proven to be challenging.^23^ The number of plants with blue-coloured flowers in the natural world is limited, accounting for 15% to 20% of angiosperms.^24^*F3ʹ5ʹH*, known as the ‘blue gene’, encodes a key enzyme in the pigmentation of blue flowers.^25^ DFR and ANS catalyse the formation of coloured anthocyanins from different substrates.^26^ Ultimately, UFGT converts anthocyanins into stable anthocyanidins.^27^ The flowers of *P. cordata* are violet (a kind of blue). Therefore, *P. cordata* is an ideal species for identifying the genes involved in anthocyanin synthesis and breeding blue-flowered plants.

In this study, we assembled the first complete genome of *P. cordata* using PacBio sequencing, Illumina sequencing, and Hi-C scaffolding technologies. To reveal the mechanisms underlying the rapid adaptation of *P. cordata*, we explored the whole-genome duplication (WGD) events, gene contraction and expansion, gene duplication, and positive selection within *P. cordata*. In addition, we compared the number of RGAs and TFs in *P. cordata* with that in other aquatic/wetland plants. Finally, we performed transcriptome analysis to investigate the mechanism of flower colour variation in *P. cordata*. Overall, we aimed to provide a valuable genomic resource and analyse the mechanisms of adaptation and colour variation in *P. cordata*.

## 2. Materials and methods

### 2.1. Plant materials and genome sequencing

**Fig. 1.**
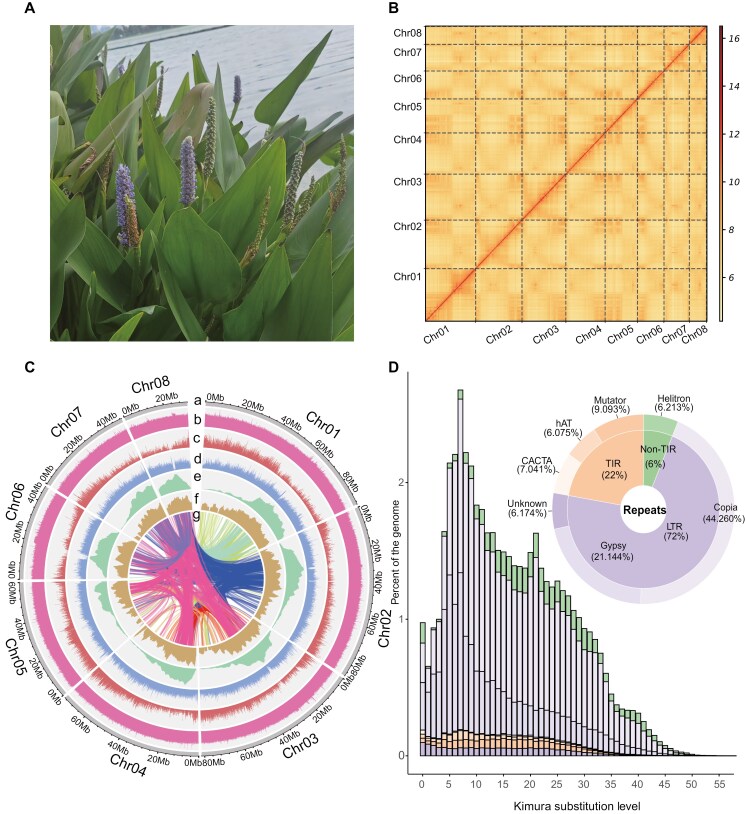
A photo of *Pontederia cordata* and characteristics of its genome. a) Leaves and flowers of *P. cordata*. b) Contact map of Hi-C-based intrachromosomal interactions. c) An overview of the genomic features of *P. cordata*. a) Circular representation of the pseudochromosomes (Mb), b) distribution of GC content, c) gene density, d) repeat density, e) LTR density, f) distribution of LAI score, g) paralogous gene pairs. All distributions were drawn in a window size of 1 Mb. d) A pie chart summarizing the TE content of the genome assembly. Histograms showing the distribution of Kimura substitution levels among the TEs. The Kimura substitution level (%) for each copy compared with its consensus sequence was used as a proxy for the expansion history of TEs.

*P. cordata* was collected from Minghu, a natural lake on the campus of China Pharmaceutical University, Nanjing, China (Fig. 1a). Leaves were used for genomic DNA extraction, while leaves and roots were used for total RNA extraction. Tissues used for PacBio HiFi, Illumina, and HiC sequencing were all obtained from the same individual. Libraries were constructed according to the manufacturer’s standard protocol (Novogene Co. Ltd., Tianjing, China). A HiFi SMRTbell library, with a 20 kb insert size, was sequenced on the PacBio Sequel II platform (Pacific Biosciences, USA). For Illumina sequencing and Hi-C sequencing, the NovaSeq 6000 platform (Illumina Inc., USA) was utilized, employing 150 bp paired-end reads. See Table S1 for sequencing data generated in this study.

### 2.2. Genome survey and genome assembly

The *K-mer* frequency was determined using Jellyfish (v2.3.0),^28^ and the genome size, heterozygosity rate, and repeat content were subsequently predicted using findGSE (v1.94. R).^29^ HiFi data were assembled using software Hifiasm, Hicanu, Peregrine, NextDenovo, and Pbipa to produce the haplotype-resolved assembly. Subsequently, the redundant haplotypes were removed using Purge_Dups (v1.2.5)^30^ and Purge Haplotigs (v1.1.1).^31^ Hi-C reads were aligned to the assembly using the Juicer pipeline (v1.60).^32^ Then, the 3D-DNA pipeline^33^ was used to construct a chromosomal-level genome. The genome was polished using Juicebox Assembly Tools (v1.11.08).^34^ Benchmarking Universal Single-Copy Orthologs (BUSCO, v5.2.1),^35^ Merqury (v1.3),^36^ and the long terminal repeat (LTR) Assembly Index (LAI, v2.9.0) were used to assess the accuracy and completeness of the genome assembly. See Table S2 for software used for genome assembly.

### 2.3. Repeat annotation and noncoding gene annotation

We used EDTA (v1.9.7),^37^ an automated software, for *de novo* repeat prediction of *P. cordata*. Transposable elements (TEs), not classified by EDTA, were further analysed using DeepTE.^38^ The Kimura substitution rates of TEs were calculated using the Perl script ‘calcDivergenceFromAlign.pl’ bundled in RepeatMasker (v4.1.5).^39^ tRNAs were annotated using tRNAscan-SE (v2.0.6)^40^ with default parameters. rRNAs and other noncoding RNAs were annotated by Infernal (v1.1.4)^41^ using the Rfam 14.1 database.^42^

### 2.4. Gene prediction and gene functional annotation

Genes were identified by integrating predictions from transcriptome-based, homology-based, and *de novo*-based methods. Nine gene prediction software (Table S3), such as PASA (v2.4.1)^43^ (based on transcriptome), GeneWise (v2.4.1)^44^ (based on homology), and AUGUSTUS (v2.4.1)^45^ (based on *de novo*), were used to predict protein-coding genes. The results of these analyses were evaluated based on the completeness of the gene and the transcriptome alignment rate.

Gene functional annotation was carried out using peptide sequences (PEPs) via 3 strategies: (1) gene ontology (GO) annotation: EggNOG-mapper (v2.1.4-2)^46^ and InterProScan (v5.52-86.0).^47^ (2) Kyoto Encyclopedia of Genes and Genomes (KEGG) annotation was carried out using GFAP.^48^ (3) Nonredundant (NR) annotation: PEPs were aligned to the NR database (https://ftp.ncbi.nlm.nih.gov/blast/db/FASTA/nr.gz, assessed on December 2023) with Diamond (v2.0.8.146).^49^ The Diamond blast results were filtered using custom scripts. Results of three strategies were integrated to form the final geneannotation of *P. cordata.*

We also used the online PlantRegMap^50^ (https://plantregmap.gao-lab.org/) to identify TFs in the *P. cordata* genome and 2 other wetland plants (*Acorus americanus* and *Acorus calamus*) and 5 aquatic plants (*Lemna minuta*, *Pistia stratiotes*, *Spirodela polyrhiza*, *Wolffia Australiana*, and *Zostera marina*) (Table S4) in monocots. Plant disease resistance-related genes (R-genes) were predicted by the RGAugury pipeline.^51^ The default *P* value cut-off for initial RGA filtering was le-5 for BLASTP. Four classes of RGAs were analysed: nucleotide-binding site (NBS)-encoding proteins, receptor-like proteins (RLPs), receptor-like protein kinases (RLKs), and transmembrane coiled-coil proteins (TM-CCs).

### 2.5. Gene duplication and WGD analysis

Duplication modes of gene pairs in *P. cordata* were identified using the DupGen_finder pipeline.^52^ In brief, *Aristolochia fimbriata* was selected as the outgroup. An all versus all BLASTP was performed to identify all possible homologous gene pairs. Then, the types of duplicated genes were annotated using MCScanX.^53^ Finally, we calculated the *Ka* (number of substitutions per nonsynonymous site), *Ks* (number of substitutions per synonymous site), and *Ka*/*Ks* values for duplicate pairs using KaKs_Calculator (v2).^54^

To identify the WGD events, we used the WGDI^55^ pipeline. The predicted proteins of *P. cordata* were subjected to BLAST searches against themselves, followed by a collinearity analysis. The *Ks* for genes in paired collinear gene groups was calculated, and the *Ks* peak was detected. For comparison, the same processes were applied to *Typha angustifolia* and *Canna edulis.* Finally, the ancestral monocot karyotype, except for that of Acoraceae (AMK-A), was employed to probe the evolutionary history, including diversification and polyploidization processes.^56^

### 2.6. Phylogenomic and gene family evolution analyses

The protein sequences of *C. edulis*, *C. indica*, *Costus pulverulentus*, *Ensete glaucum*, *Musa acuminata*, *P. cordata*, *Thalia dealbata*, *Z. officinale*, and *Oryza sativa* were retrieved from public databases (Table S4) and used for phylogenomic analysis. Orthologous genes among these genomes were detected using OrthoFinder (v2.5.5)^57^ with default parameters. We used MAFFT (v7.520)^58^ to align the protein sequences of single-copy orthologous genes and converted the alignments into codon sequences via PAL2NAL (v14.1.56).^59^ We used trimAl (v1.4.rev22)^60^ to remove non-conserved or divergent regions, and then we used RAxML-NG (v1.2.0)^61^ to construct the phylogenetic trees. The divergence time was estimated using the MCMCtree of PAML (v4.10.5)^62^ with the following calibration points: *Z. officinale* and *C. pulverulentus* (47.5–77.7) million years ago (Mya) and *E. glaucum* and *M. acuminata* (45.1–69.1) Mya. Two additional calibrated points were obtained from Timetree (http://www.timetree.org, accessed on December 2023). CAFE5 (v1.1)^63^ was used to evaluate the expansion and contraction of the gene families in each lineage. KEGG enrichment analysis for expanded or contracted gene families was performed using the R package ClusterProfiler.^64^

### 2.7. Identification of anthocyanin gene family members

The reference sequences of *AtPAL*, *AtC4H*, *At4CL*, *AtCHS*, *AtCHI*, *AtF3H*, *AtFLS*, *AtDFR*, *AtANS*, and *AtANR* were downloaded from The Arabidopsis Information Resource (TAIR) database. As the *F3ʹ5ʹH* and *UFGT* genes are unavailable from *A. thaliana*, sequences of the two genes were downloaded from the Universal Protein Knowledgebase (UniProt) database (Table S5). At least one sequence of each gene was used as a query to search against the proteins of *P. cordata* with BLASTP. Finally, the candidate genes were filtered using PFAM domains.

The physicochemical properties of genes in anthocyanin biosynthesis in *P. cordata* were analysed using ExPASy ProtParam (http://www.expasy.org/tools/protparam.html). Subcellular localization was predicted using CELLO (http://cello.life.nctu.edu.tw) and WoLF PSORT (https://wolfpsort.hgc.jp). The gene structure and chromosomal localization of each gene were analysed by TBtools software (v2.034).^65^ The MEME online program (http://meme-suite.org/tools/meme) was used to analyse conserved motifs of these genes. The cis-acting elements of the genes were analysed using PlantCARE (http://bioinformatics.psb.ugent.be/webtools/plantcare/html/).

### 2.8. Transcriptomic analysis

To investigate the differentially expressed genes (DEGs) between violet corolla tissues and white corolla tissues, transcriptome sequencing was carried out for 3 samples of violet corolla tissues and 3 samples of white corolla tissues from *P. cordata*. Total RNA was extracted and eluted in 50 μL of RNase-free water per reaction, and RNA quality was determined using an Agilent 2100 Bioanalyzer (Agilent Technologies, Santa Clara, USA). Then, 150 bp PE mRNA sequencing libraries were prepared and sequenced on the DNBSEQ-T7 platform (MGI, Wuhan, China). Low-quality bases and adapter sequences were removed using fastp (v0.23.2).^66^ Clean reads were mapped against the final genome assembly by HISAT2 (v2.1.0).^67^ Uniquely mapped reads were used to calculate the counts and transcripts per kilobase million (TPM) by StringTie (v2.2.1).^68^ DEGs between the 2 groups of samples were inferred using DEseq2,^69^ with a |log_2_-fold change (FC)| > 1.5 and a *P* value < 0.05. Based on the STRING database (https://cn.string-db.org/), the protein–protein interaction (PPI) was analysed with *Arabidopsis thaliana* as a reference, and a plot was made with Cytoscape (v3.8.2).^70^ Finally, ClusterProfiler was used to analyse the enrichment of GO and KEGG functions of the DEGs. Finally, we quantified these genes in four parts- stem, leaf, white flower, and violet flower- and the heatmap and motif were visualized using TVBOT (https://www.chiplot.online/tvbot.html).^71^

## 3. Results

### 3.1. Chromosome-scale genome assembly of *P. cordata*

For the genome assembly of *P. cordata*, we generated 68 Gb of Illumina reads (2 × 150 bp), 9.6 Gb of PacBio HiFi reads (with an N50 length of 15.70 kb), and 136 Gb of Hi-C reads (Table S1). *K-mer* analyses indicated that the genome was approximately 512.6 Mb in size, with 44.1% repeats and 0.72% heterozygosity (Fig. S2). The genome assembly using PacBio HiFi reads generated 378 contigs with an N50 of 3.88 Mb and a size of 540 Mb (Table S2). Using Hi-C reads, 527.5 Mb (97.50%) of the contigs were anchored and ordered into 8 pseudochromosomes (Fig. 1b). The final genome assembly has a BUSCO completeness score of 98.6% (Fig. S3) and a quality value (QV) of 52.1 (Table S2). The QV is similar to that of other genome assemblies, such as *Astragalus membranaceus* (QV:48.58),^72^*Fallopia multiflora* (QV:51.42).^73^ This means our genome assembly is highly accurate. In addition, the final assembly has an average LAI of 16.73, which satisfied the ‘reference’ quality criterion (10 ≤ LAI < 20) (Fig. 1c) in Commelinales.^74^

### 3.2. Transposable elements of *P. cordata*

Repeats in the genome of *P. cordata* were identified by integrating predicted repeats from homology-based and *ab initio*-based methods. We found that TEs were major components (53.44%) within the *P. cordata* genome. Among these TEs, the main type was long terminal repeat-retrotransposons (LTR-RTs), which accounted for 36.29% (22.44% of *Copia*, 10.72% of *Gypsy*, and 3.13% of unknown) of the *P. cordata repeats*. The remaining repeats included terminal inverted repeat (TIR), non-long terminal repeat (nonTIR), and non-long terminal repeat (nonLTR), which accounted for 12.09%, 3.15%, and 0.07% of the genome, respectively (Table S6).

To examine the evolution of TEs in *P. cordata* genome, we estimated Kimura substitution levels by comparing TE copies and their consensus sequences (Fig. 1d). Assuming that TEs are under neutral conditions, the older a TE family is, the more mutations it may contain.^75^ The landscape of Kimura substitution levels showed that the divergence for most TE sequences was low (5% to 30%). We also estimated the insertion time of intact LTR-RTs. Approximately 63.2% of the intact LTR-RTs were younger than 1 million years (Table S7), with peak insertion times of 0.76 and 0.22 Mya for the *Copia* and *Gypsy* elements (Fig. S4). The results indicated a recent expansion of LTRs in *P. cordata*.

### 3.3. Genome annotation of *P. cordata*

Among the different software for gene prediction, GETA excelled in terms of both the completeness of gene prediction and the transcriptome alignment rate (Fig. S5, Table S3). GETA identified 29,389 protein-coding genes with an average length of 4,691 bp. The proportion of completely covered and partially covered BUSCO genes was 92.1% (Table 1). Twenty-nine thousand three hundred and thirty-eight of the 29,389 protein-coding genes (99.82%) were annotated to the Gene Ontology (GO), KEGG, EggNOG, and Nonredundant (NR) databases using custom scripts. The annotated noncoding RNAs included 159 microRNAs (miRNAs), 1,017 small nuclear RNAs (snRNAs), 608 transfer RNAs (tRNAs), and 168 ribosomal RNAs (rRNAs) (Table S8).

**Table 1:** Statistics for the assembly and annotation of *P. cordata*.

Feature	*P. cordata*
Genome estimate	
Genome size (Mb)	512.6
Heterozygosity rate (%)	0.72
Chromosome number	8
Genome assembly	
Assembly size (Mb)	527.5
Number of contigs	234
Contig N50 (Mb)	5.23
Number of scaffolds	8
Scaffold N50 (Mb)	48
BUSCOs (%)	98.6
Repeat region % of assembly	53.44
LAI	16.73
Genome annotation	
Protein-coding genes	29389
Average gene length (bp)	4691.15

### 3.4. Phylogenetic analysis and divergence time estimation of *P. cordata*

**Fig. 2.**
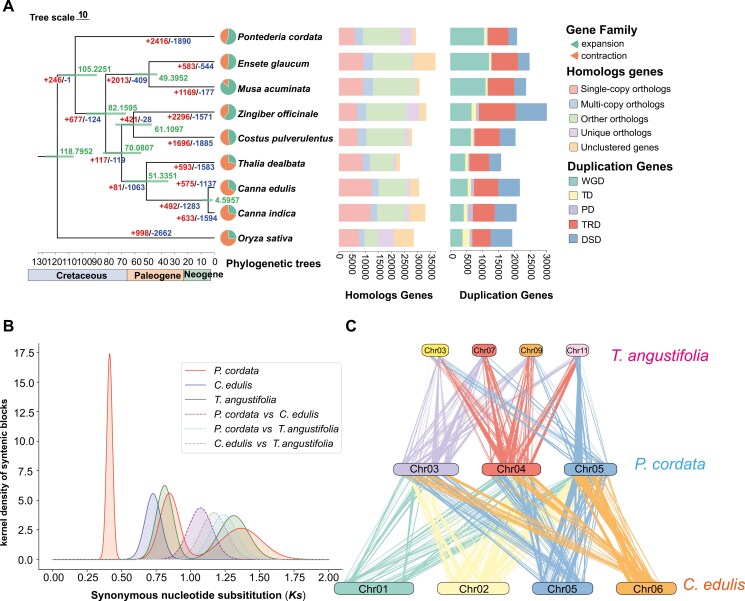
Phylogenomic analyses and whole-genome duplication analyses. a) The numbers of gene family expansion and contraction events are indicated by left and right numbers on each branch, respectively. The divergence times (Mya) are indicated using the green numbers beside the branch nodes. Pie charts show the proportions of gene families that experienced expansion or contraction. The stacked bar chart represents the distribution of homologous genes in each species, such as single-copy, multiple-copy, and unique paralog genes and other orthologs. The distribution of duplicated genes included WGD, tandem duplicates (TD), proximal duplicates (PD), transposed duplicates (TRD), and dispersed duplicates (DSD). b) Distribution of *Ks*, which represented the Gaussian fit of the raw *Ks* counts of orthologous and paralogous genes among *P. cordata*, *T. angustifolia*, and *C. edulis*. c) Synteny blocks between the *T. angustifolia*, *C. edulis*, and *P. cordata* genomes.

To investigate the phylogenetic position of *P. cordata*, we used genomes of *P. cordata*, 7 Zingiberales species, and 1 outgroup (*O. sativa*) (Table S4). A total of 19,719 orthologous families and 2,310 single-copy families were identified among these species. Based on the 2,310 single-copy genes, a maximum likelihood-based phylogenetic analysis was conducted (Fig. 2a). *P. cordata* was sister to a clade comprising Zingiberales species. The divergence time between Commelinales and Zingiberales was estimated to be ca. 105.2 Mya.

### 3.5. WGD in *P. cordata*

To explore WGD events in *P. cordata*, we conducted syntenic analysis for *P. cordata*, *T. angustifolia*, and *C. edulis* using the WGDI pipeline (Fig. 2B, Fig. S6, Table S4). The analysis of collinearity and *Ks* distribution in *P. cordata* revealed 3 distinct peaks, which are indicative of 3 WGD events (Fig. S6). Two peaks were observed at *Ks* = 0.413 and *Ks* = 0.846, and there were 1:4 syntenic blocks between *T. angustifolia* and *C. edulis* (Fig. 2c, Fig. S7), which indicated that *P. cordata* recently experienced 2 independent genome-wide replication events. A peak at *Ks* = 1.37 was observed before their divergence, accompanied by 1:8 syntenic blocks with AMK-A (Fig. S7), indicating an ancient WGD event, consistent with the tau (*τ*) WGD and had been reported in Commelinid.^76^ The peak of *Ks* = 0.728 indicated that *C. edulis* experienced recent whole-genome triplication (WGT) (Fig. S6). *T. angustifolia* has experienced 2 genome-wide replication events: genome-wide tripling (*Ks* = 0.808), which is sigma (*σ*) WGT and was reported in Poales,^77^ and *τ* WGD (Figs. S6 and 7).

### 3.6. Gene duplication associated with environmental adaptation in *P. cordata*

The duplication model for 24,536 of the 29,389 genes in *P. cordata* genome was annotated. The duplication modes were classified into 5 categories (Figs. S8 and S9), and the majority of the duplicated genes were attributed to WGD (45.36%).

**Fig. 3.**
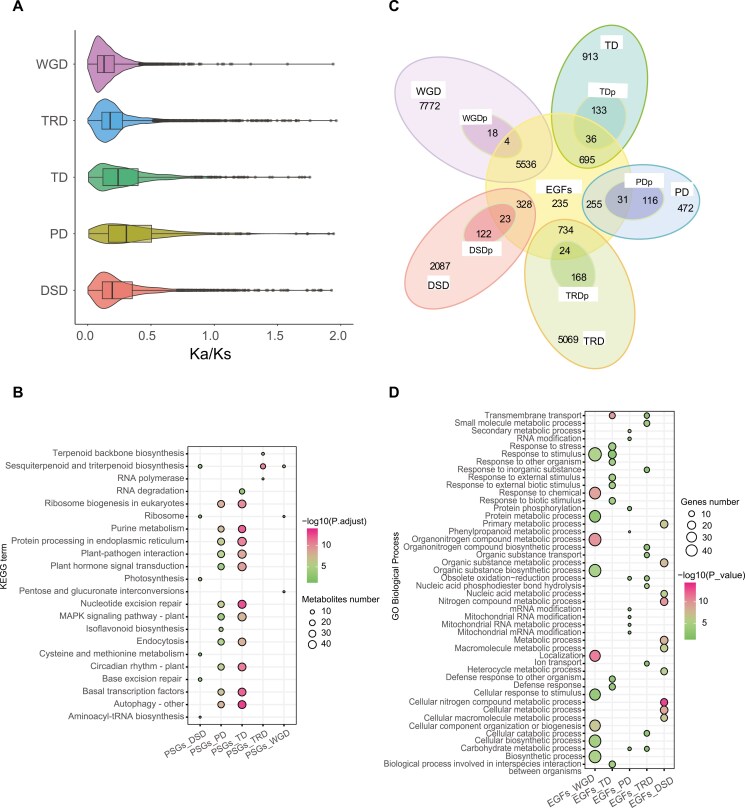
Evolutionary pressures and functional enrichment of duplicated genes in *P*. *cordata*. a) *Ka*/*Ks* ratio distributions of gene pairs derived from different types of duplications. b) KEGG functional enrichment analysis of genes overlapping between PSGs and 5 modes of duplication. c) Venn diagram showing the possible logical relationships between members of the expanded gene family and modes of duplication. DSDp, dispersed duplication that experienced positive selection; EGFs, expanded gene families; PDp, proximal duplication that experienced positive selection; TDp, tandem duplication that experienced positive selection. d) GO enrichment of genes overlapping between expanded gene families and 5 modes of duplication. The enriched GO terms with corrected *P* values < 0.05 are presented. The colour in the circles represents the statistical significance of the enriched GO terms. The size of the circles represents the number of genes in a GO term. The GO terms of all annotated genes are provided as background information.

We calculated the *Ka/Ks* for genes derived from different duplication modes. The genes of proximal duplication (PD) and tandem duplication (TD) had a higher *Ka*/*Ks* ratio (Fig. 3a). We identified 670 duplicated genes that displayed positive selection (*Ka*/*Ks* > 1), namely, positively selected genes (PSGs). GO and KEGG enrichment analyses (Fig. S10) indicated that these PSGs were significantly enriched in ‘environmental information processing’, such as the ‘MAPK signalling pathway-plant’, and ‘plant-pathogen interaction’ (Fig. 3b, Table S9).

Analysis of gene family expansion and contraction revealed 7,901 expanded and 1,074 contracted genes in *P. cordata*. The majority of the expanded genes (70.07%) originated from WGD (Fig. 3c, Fig. S11). GO analyses indicated that the expanded genes were enriched in divergent functions (Fig. 3d, Fig. S12). For example, the expanded gene families derived from WGD were mainly enriched in ‘metabolism’ and ‘exosome’. Nevertheless, expanded gene families derived from TD were mainly enriched in ‘response to oxidative stress’ and ‘defense response to bacterium’.

### 3.7. RGAs and TFs associated with environmental adaptation in *P. cordata*

**Fig. 4.**
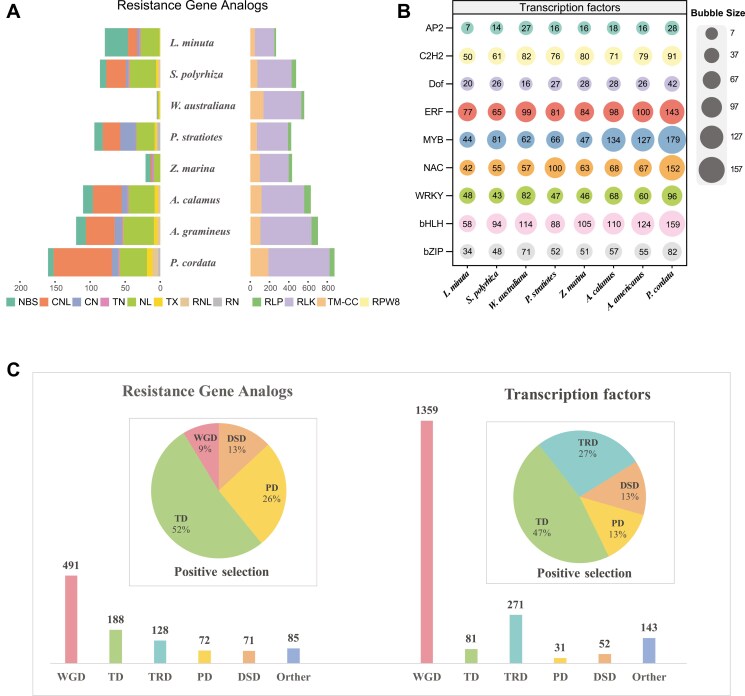
Resistance genes and TFs in *P*. *cordata.* a) Histogram shows the number of RGAs in 8 aquatic plants. b) The bubble matrix shows 9 TFs involved in environmental adaptation in the 8 aquatic plants. The numbers in the bubbles represent the number of TFs in each species. c) Bar charts showing the numbers of RGAs and TFs related to duplication events. Pie charts show the proportions of duplication types among the positively selected RGAs and TFs.

We identified 1,035 RGAs in *P. cordata*, which are classified as NBS-encoding genes (160), RLPs (51), RLKs (637), and TM-CCs (185). Compared to the other 6 aquatic/wetand plants, *P. cordata* has a greater number of RGAs (Fig. 4a). This increase was particularly evident in CNLs (NBS-encoding genes) and RLKs (receptor-like kinases).

In addition, we identified a total of 1,937 TFs in *P. cordata*, a number that exceeds the counts observed in other aquatic/wetland plants (Fig. 4b). For example, *L. minuta* had 808 TFs and *S. polyrhiza* had 1,048 TFs, despite they have a similar number of total genes as *P. cordata*. Notably, an increased number of TFs, including *MYB*, *WRKY*, *bHLH*, *NAC*, *ERF*, *AP2*, and *C2H*2, were detected in *P. cordata* across various gene families. For example, we identified 179 *MYBs* in *P. cordata*, more than that in other aquatic monocot species such as *Z. marina* (44 genes), *S. polyrhiza* (81 genes), and *P. stratiotes* (66 genes).

Analyses found that approximately 491 RGAs (47.4%) and 1359 TFs (70.2%) were from WGD (Fig. 4c). In addition, approximately 12 (52%) of the positively selected RGAs and 14 (47%) of the positively selected TFs were identified as tandem duplicated genes (Fig. 4d). Notably, TD exhibited a greater frequency of positive selection in response to environmental pressures.

### 3.8. Analyses of flower colour variation in *P. cordata*

We identified 82 genes associated with anthocyanin biosynthesis in *P. cordata* (Table S10, Fig. S13), including *PAL* (7), *C4H* (8), *4CL* (36), *CHS* (11), *CHI* (2), *F3H* (1), *F3*ʹ*5*ʹ*H* (6), *DFR* (5), *ANS* (3), and *UFGT* (3). These genes were distributed on 8 chromosomes (Fig. S14), with the highest number on chromosomes 2 (21 genes) and 3 (17 genes), and the lowest number on chromosome 6 (2 genes). These genes are divided into different duplication types, with WGD accounting for 40% and TD for 23% of the total gene duplications (Table S10). For example, all repeated copies of *C4H* are derived from WGD, and 8 of 11 *CHS* genes are caused by TD. Subcellular localization analysis revealed that most of the genes were expressed in the chloroplast, cytoplasm, and plasma membrane. The physicochemical properties and conserved motifs of these genes were similar to those of the reference genes (Table S11, Fig. S15), which also confirmed the reliability of these candidate genes.

**Fig. 5.**
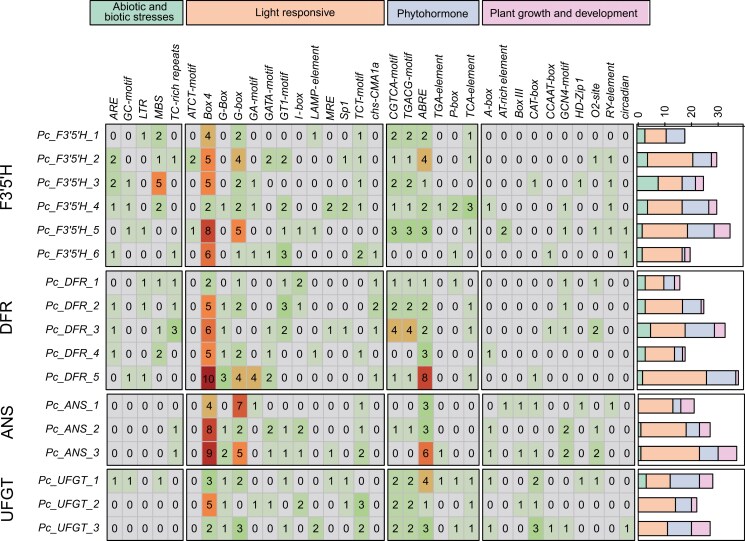
Classification and statistics of cis-acting elements for genes in anthocyanin biosynthesis pathway. The 34 kinds of cis-acting elements were divided into 4 categories. The number in the grid represents the number of elements. The stacked bar chart on the right represents the number and distribution of the 4 categories of cis-acting elements.

Cis-acting elements are crucial for regulating the initiation and efficiency of gene transcription.^78^ In this study, a 2,000 bp sequence upstream of each gene was analysed to predict cis-acting elements in 17 copies of *F3*ʹ*5*ʹ*H*, *DFR*, *ANS*, and *UFGT,* which are important for anthocyanin production (Table S12). A total of 33 cis-acting elements were identified within the promoter region and categorized into 4 primary groups: abiotic and biotic stresses elements (44), light-responsive elements (234), phytohormone-responsive elements (117), and plant growth and development elements (55) (Fig. S16). Within the promoter region (Fig. 5), the groups of light-responsive elements were the most prevalent among all identified cis-acting elements. All the genes contained Box 4 cis-acting elements, which play an important role in the biosynthesis of anthocyanins.^79^

**Fig. 6.**
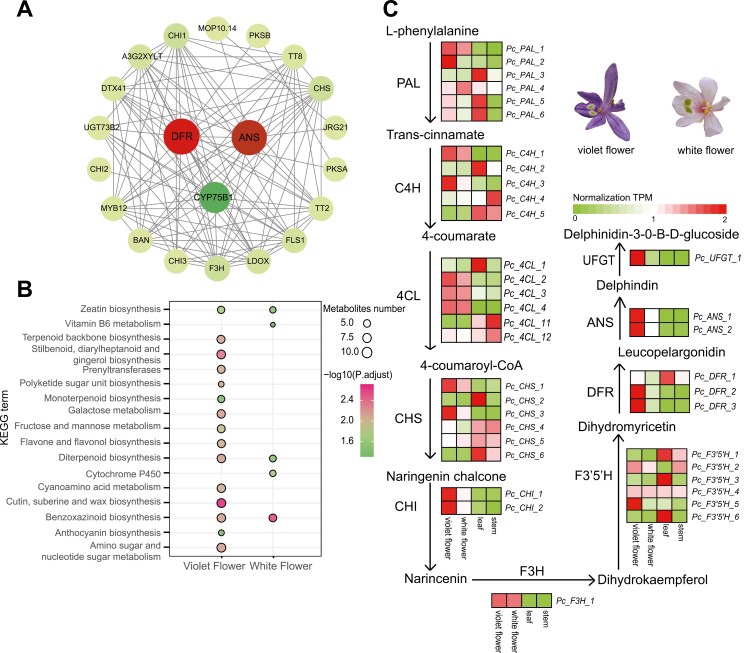
Expression levels of genes in the anthocyanin pathway of *P. cordata*. a) Protein‒protein interaction (PPI) of up-regulated genes in purple flowers, with larger circles indicating stronger correlations. b) KEGG enrichment analysis of up-regulated genes in violet-coloured and white-colored flowers. c) Anthocyanin biosynthetic pathway and expression profiles of genes in the pathway. Schematic representation of anthocyanin biosynthesis and the expression profiles of the enzyme-encoding genes. From left to right, the squares represent purple flowers, white flowers, leaf, and stem tissues, respectively. ANS, anthocyanidin synthase; C4H, cinnamate-4-hydroxylase; 4CL, 4-coumarate CoA ligase; CHS, chalcone synthase; CHI, chalcone isomerase; DFR, dihydroflavonol-4-reductase; F3H, flavanone 3-hydroxylase; F3ʹ5ʹH, flavonoid-3ʹ,5ʹ-hydroxylase; PAL, phenylalanine ammonia-lyase; UFGT,3-O-glucosyltransferase.

To investigate colour variations in *P. cordata*, we utilized transcriptomic data to evaluate differential gene expression between violet and white flowers (Fig. S17). A total of 367 DEGs were identified, with 239 genes up-regulated in violet flowers and 128 genes up-regulated in white flowers (Fig. S18). Analysis of the PPI network (Fig. 6a, Table S13) revealed that up-regulated genes associated with anthocyanin biosynthesis in violet flowers were clustered, with *DFR*, *ANS*, and *CYP75B1* exhibiting the most significant correlations. KEGG enrichment analysis revealed that these genes were predominantly enriched in the ‘flavone and flavonol biosynthesis’ and ‘anthocyanin biosynthesis’ pathways. These results indicated that the variation in flower colour of *P. cordata* was caused by DEGs involved in anthocyanin biosynthesis (Fig. 6b, Table S14). Subsequently, we discovered that downstream biosynthetic genes, including *F3ʹ5ʹH*, *DFR*, *ANS*, and *UFGT*, are more highly expressed in violet flowers than in white flowers, as well as in leaf and stem tissues (Fig. 6c, Table S15). The elevated expression of these genes in violet-coloured flowers promotes the accumulation of anthocyanins, which is responsible for their characteristic violet coloration. Conversely, in white flowers, the expression of the *DFR* and *UFGT* genes is nearly negligible, preventing the formation and stabilization of coloured anthocyanins.

## 4. Discussion

Genomics has played an increasingly important role in revealing biodiversity, species evolution,^80,81^ and breeding.^82^ The Commelinales, as an important branch of the commelinids, contains not only a variety of unique ornamental plants (such as *Anigozanthos flavidus* and *Tradescantia sillamontana*), but also some invasive weeds (such as *Commelina communis*, *Tradescantia fluminensis*, and *Pontederia crassipes*). These weeds have caused ecological damage and economic loss to some areas.^83,84^ However, due to the lack of genomic resources of the Commelinales, the molecular mechanisms of how these species rapidly adapt to new habitats are rarely revealed. To date, only one species (*Pontederia crassipes*^85,86^) in the Commelinales has had its whole been sequenced. In this study, we utilized a combination of Illumina, HiFi, and Hi-C technology to generate a chromosome-level genome of *P*. *cordata*. The length of *P*. *cordata* genome was 527.5 Mb and it was anchored on 8 pseudochromosomes, with 98.6% complete BUSCO value and 29,389 predicted genes. Compared with the BUSCO value of other species in commelinids, the genome of *P*. *cordata* showed good completeness (Fig. S2). Therefore, the *P*. *cordata* genome will be helpful for studying the molecular mechanisms by which plants in the Commelinales adapt to their environment.

By utilizing a dataset comprising 2,310 single-copy orthologous genes across eight species, we constructed a phylogenetic tree that positions *P*. *cordata* as sister to a clade formed by Zingiberales species. This finding is consistent with APG IV^87^ and underscores the utility of single-copy genes in elucidating accurate phylogenetic relationships.

Our results indicated that the majority of TEs in the *P*. *cordata* genome show low Kimura substitution levels, suggesting a recent or ongoing expansion of these elements within the genome. This is supported by the insertion times (0.76 and 0.22 Mya for the *Copia* and *Gypsy* elements, respectively) for intact LTR-RTs. It is known that a species’ ability to successfully invade a new habitat largely depends on the number and nature of genetic variants available for selection during invasion process.^88^ Previous studies have shown that the insertion of TEs can produce new genetic variants and affect the expression of neighbouring genes in response to environmental changes, thereby enhancing environmental adaptability. For instance, in the *A. thaliana* genome, retrotransposon insertion in *FLC* gene may enhance adaptation to high-concentration herbicide environments.^89^ In addition, the insertion of a transposon into the promoter region of the *UBC12* gene in japonica rice has led to an up-regulation of its expression, which in turn has improved the rice’s ability to germinate at low temperatures.^90^ The recent expansion of LTRs in *P*. *cordata* may have contributed to the generation of novel genetic variants and influence gene expression and regulation, thereby helping the species break through the genetic bottleneck and potentially driving its adaptation to new environments.

WGD has long been recognized as an important process that contributes to adaptive evolution and diversification.^91^ We found that the commelinid shared an ancient WGD event, which is consistent with previous studies.^76^ In addition, we revealed two WGD events occurred in Commelinales. However, due to the limited number of genomic data in Commelinales, the exact positions of the 2 WGD events remain unknown. Further studies need to explore the positions.

We found that WGD accounts 47.6% of the duplicated genes in *P*. *cordata* genome (Fig. 2A). WGD increases gene redundancy, enhancing gene diversity, and providing raw materials for new functions and optimizing existing ones.^52^ In addition, we found that WGD was the major contributor for the expanded gene families in *P*. *cordata* genome, recovered from CAFE (Fig. 3c). GO analysis showed that expanded gene families derived from TD were mainly enriched in ‘response to oxidative stress’ and ‘defense response to bacteria’. Similarly, TD and PD events led to the expansion of genes involved in pathogen defense, stress response, and nutrient acquisition in the aquatic plant *Lemna minuta*.^18^ The gene family expansion may have increased the number and diversity of genes, providing additional evolutionary resources that help *P. cordata* adapt to environmental changes and new ecological niches.^92,93^

We observed that duplicated genes derived from PD and TD showed a higher *Ka*/*Ks* ratio, indicating these genes experienced more rapid functional divergence and stronger positive selection pressures than genes deriving from other duplication modes.^82^ The enrichment analysis showed that PSGs (PD and TD) were significantly enriched in ‘environmental information processing’ (Fig. 3B). Previous studies reported that these enrichment terms are associated with environmental adaptability, particularly related to plant disease resistance.^94,95^ The positive selection may enhance the ability of *P*. *cordata* to adapt to environmental conditions, which is crucial for its survival and reproduction in a variable environment.

RGAs play critical roles in plant–pathogen interactions in the plant immune system.^13,96^ We identified a significant number of RGAs in *P*. *cordata*. WGD was the primary driver accounting for the expansion of RGAs, which is consistent with the main reason for the diversification of wheat RGAs.^97^ It is worth noting that TD is another major reason for the expansion of RGAs in *P*. *cordata.* TD-driven expansion of RGAs has also been observed in some other aquatic plants, such as *Nymphoides indica*,^4^*Pistia stratiotes*,^20^ and *Spirodela polyrhiza*.^98^ Interestingly, a reduction in RGAs was observed in *P*. *crassipes*. *P*. *crassipes* may achieve invasion through rapid growth and extensive reproduction,^85^ at the expense of disease resistance. This suggests that aquatic plants employ a variety of strategies to adapt to their environment.

TFs are crucial for plant growth and development.^99^ We found an increased number of TFs in *P*. *cordata* attributed to WGD, particularly the *MYB* TFs. Multiple studies have shown that *MYBs* can enhance plant tolerance to abiotic stresses such as drought by participating in the biosynthesis of metabolites such as flavonoids and cuticles.^100,101^ Through polyploidy, genes in plants might increase rapidly in a short period, providing a large amount of genetic materials, which is considered to be an important force for adaptive evolution and diversification of plants. The 2 recent WGD events in *P*. *cordata* are the main drivers for the expansion of RGAs and TFs. In addition, during its invasion of different ecological environments, *P*. *cordata* might have faced various biological and abiotic pressures,^102^ which are likely to hinder its successful invasion. For example, some microbial pathogens employ various strategies to evade plant immunity,^103^ such as masking their recognition signals or secreting effector proteins that manipulate host cell biology. The observed expansion of RGAs and TFs in *P*. *cordata* could be a response to these evasion strategies, enabling the plant to maintain or enhance its immune surveillance.

Flower colour is an important characteristic of ornamental plants, and ornamental plant varieties with novel and colourful flowers have always been an important goal of breeding. Anthocyanins are one of the main pigments contributing to a broad variety of colours in flowers.^21,104^ Our analysis indicated that genes associated with anthocyanin biosynthesis in *P*. *cordata* exhibit differential expression in flowers with different colours. The high expression levels of these genes might promote the accumulation of anthocyanins, leading to the purple floral phenotype. In contrast, the low expression levels in white flowers prevent the formation and stabilization of coloured anthocyanins, resulting in white colour. These findings are consistent with previous research reported in *Brassica oleracea*^105^ and *Primula vulgaris*,^106^ further confirming the key role of these genes in anthocyanin biosynthesis.^24–26^ In addition, the analysis of cis-acting elements reveals the importance of light-responsive elements in anthocyanin biosynthesis, which may be related to the defensive role of flower colour formation against ultraviolet radiation.^79,107^ These results not only provide insights into the molecular mechanisms underlying colour variation in *P*. *cordata* but also offer a reference for studies on colour variation in other plants.

## 5. Conclusions

This study reported the first chromosome-level genome assembly for *P. cordata*. Comparative genomic analysis indicated that *P. cordata* underwent 3 WGD events. Furthermore, KEGG analysis of duplicated genes revealed that they are involvement in plant defense mechanisms and disease resistance, thereby playing a crucial role in environmental adaptation. Compared with other aquatic monocot, *P. cordata* has more RGAs and TFs, which could enhance its rapid adaptation to the environment. We identified 82 genes in the anthocyanin biosynthesis pathway in *P. cordata*. Differential gene expression analysis revealed that the down-regulation of the *F3*ʹ*5*ʹ*H*, *DFR*, *ANS*, and *UFGT* genes may result in colour variation from violet to white in *P. cordata* flowers. Our findings offer valuable insights into the mechanisms of environmental adaptation and provide a valuable resource for genetic, environmental, and breeding studies for *P. cordata*.

## Supplementary Material

dsaf002_suppl_Supplementary_Figures

dsaf002_suppl_Supplementary_Tables

## Data Availability

All sequence data have been deposited at the NCBI under the BioProject PRJNA1045305. The genome assembly, annotations, and predicted peptides are available on FigShare at the link: https://doi.org/10.6084/m9.figshare.24866487.v1. The genome assembly also have been deposited in the Genome Warehouse in National Genomics Data Center, China National Center for Bioinformation under accession number GWHFIGH00000000.
