# Supplementary material for: Chromosome-level genome assembly of Pontederia cordata L. provides insights into its rapid adaptation and variation of flower colours
Source: DNA Res. 2025 Jan 29;32(2):dsaf002. doi: 10.1093/dnares/dsaf002 (PMC11879222; doi:10.1093/dnares/dsaf002)
Supplement: dsaf002_suppl_Supplementary_Figures [file dsaf002_suppl_supplementary_figures.docx]

Supplementary materials for “*Chromosome-level genome assembly of Pontederia cordata* L. *provides insights into its rapid* [*adaptation*](https://www.sciencedirect.com/science/article/pii/S0888754323001805) *and variation of flower colors*”

Jiale Wang^1,2,†^, Wenda Zhang^2,†^, Xiaodong Yang^1^, Puguang Zhao^2^, Xiangyu Wang^2^, Shuying Zhao^1*^, Lingyun Chen^2^

^1^School of Environment and Ecology, Jiangsu Open University, Nanjing 210036, China

^2^School of Traditional Chinese Pharmacy, China Pharmaceutical University, Nanjing 211198, China

^*^To whom correspondence should be addressed. Tel. +86-18205180786. Email: [syingwhu@126.com](mailto:syingwhu@126.com)

^†^These authors contributed equally to this work.

## Supplementary figures


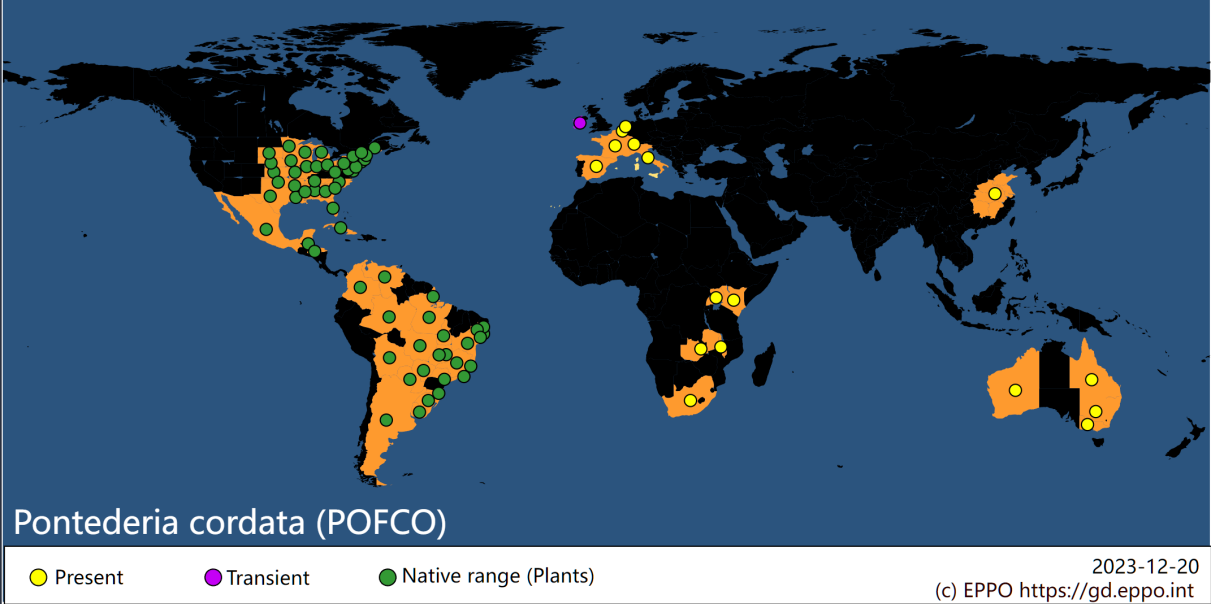


**Supplementary Figure S1.** **Global distribution of *P. cordata*** (accessed from

<https://gd.eppo.int/taxon/POFCO>, Dec 2023).

*P. cordata* is an emerging aquatic plant in Commelinales and native to the American continent. It has been introduced to many countries or regions as a horticultural plant. It colonizes shallow waters and disperses through rapid vegetative propagation. In some regions where the plant is cultivated, it has exhibited invasive behavior.


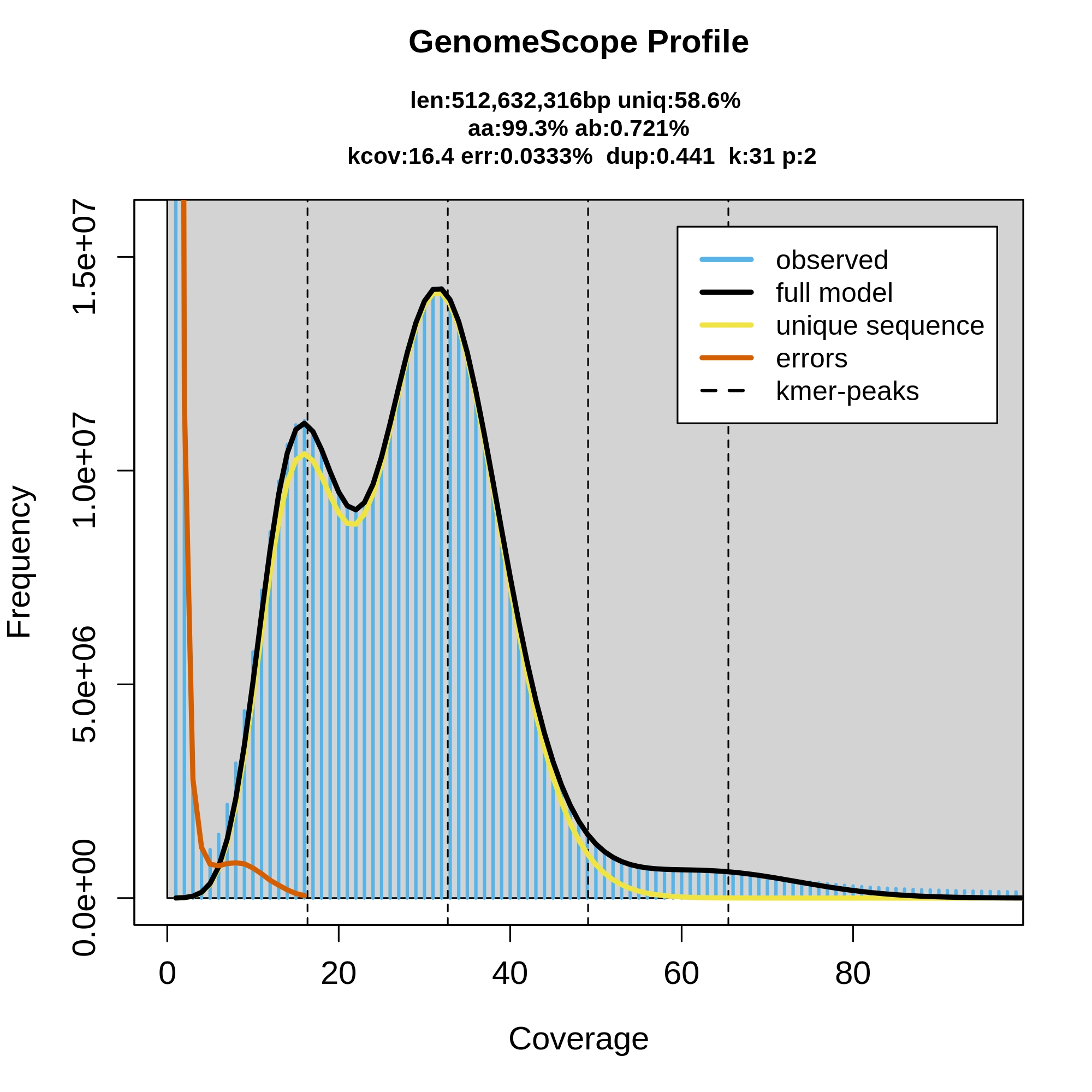


**Supplementary Figure S2.** **The *K*-mer analysis of *P. cordata* genome.**

The *K-mer* analyses indicated that the genome was approximately 512.632 Mb in size with 44.1% of repeats and 0.72% of the heterozygosity rate.

**
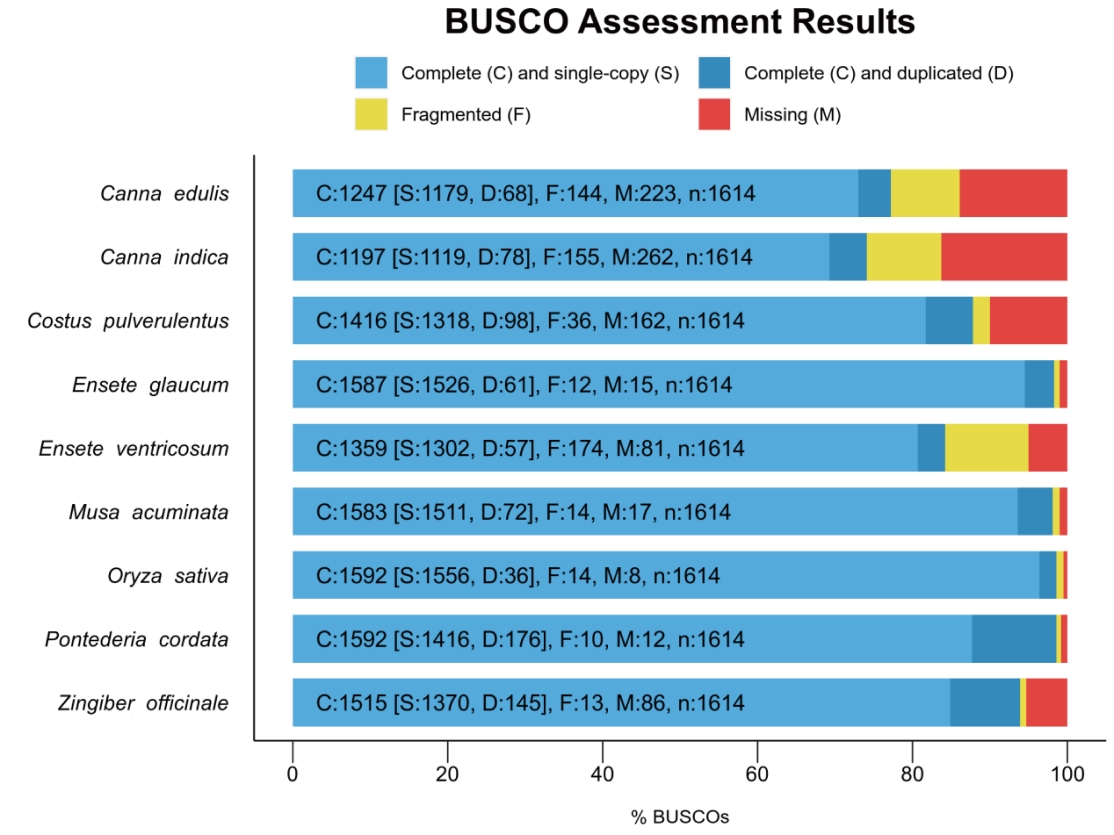
**

**Supplementary Figure S3. The BUSCO assessment of representative species in Commelinales.**

The assembled genome exhibits better performance in terms of genome integrity, as assessed by BUSCO, compared to several other species.


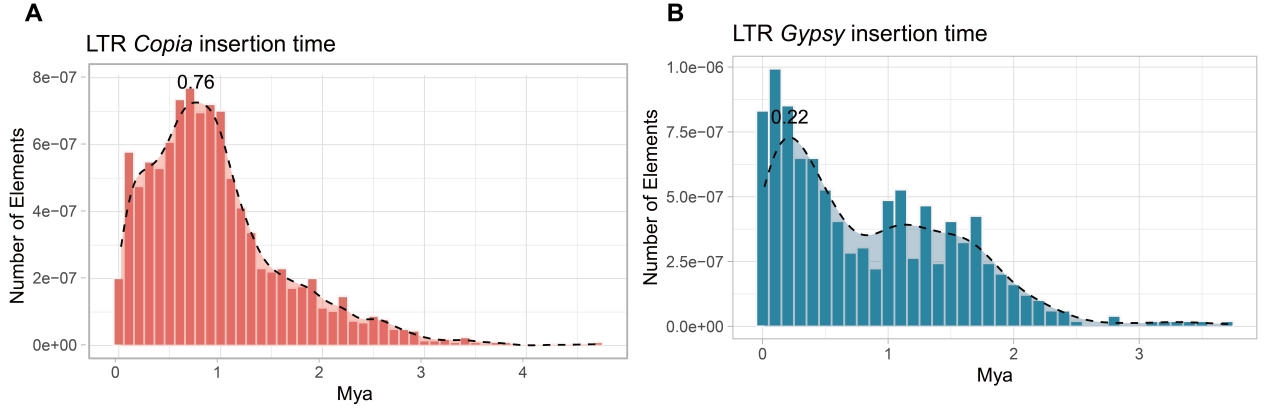


**Supplementary Figure S4.** **Distribution of insertion time of LTRs in the *P. cordata* genome.**

We found that transposable elements (TEs) were major components (53.44%) of the *P. cordata* genome. Among these TEs, the main type was the long terminal repeats (LTRs) that account for 36.29% (22.44% *Copia* and 10.72% *Gypsy*) in the *P. cordata* genome. The rest are terminal interspersed nuclear elements, DNA transposons, and long interspersed nuclear elements, which account for 12.9%, 3.15%, and 0.07% of the genome respectively (Supplementary Table S6).

We estimated the insertion time of intact LTR-RTs. Approximately 63.2% of the intact LTR-RTs were younger than one million years (Supplementary Table S7), with peak insertion times of 0.76 and 0.22 Mya for *Copia* and *Gypsy* elements, respectively. The result indicated a recent expansion of LTR in *P. cordata.*

**
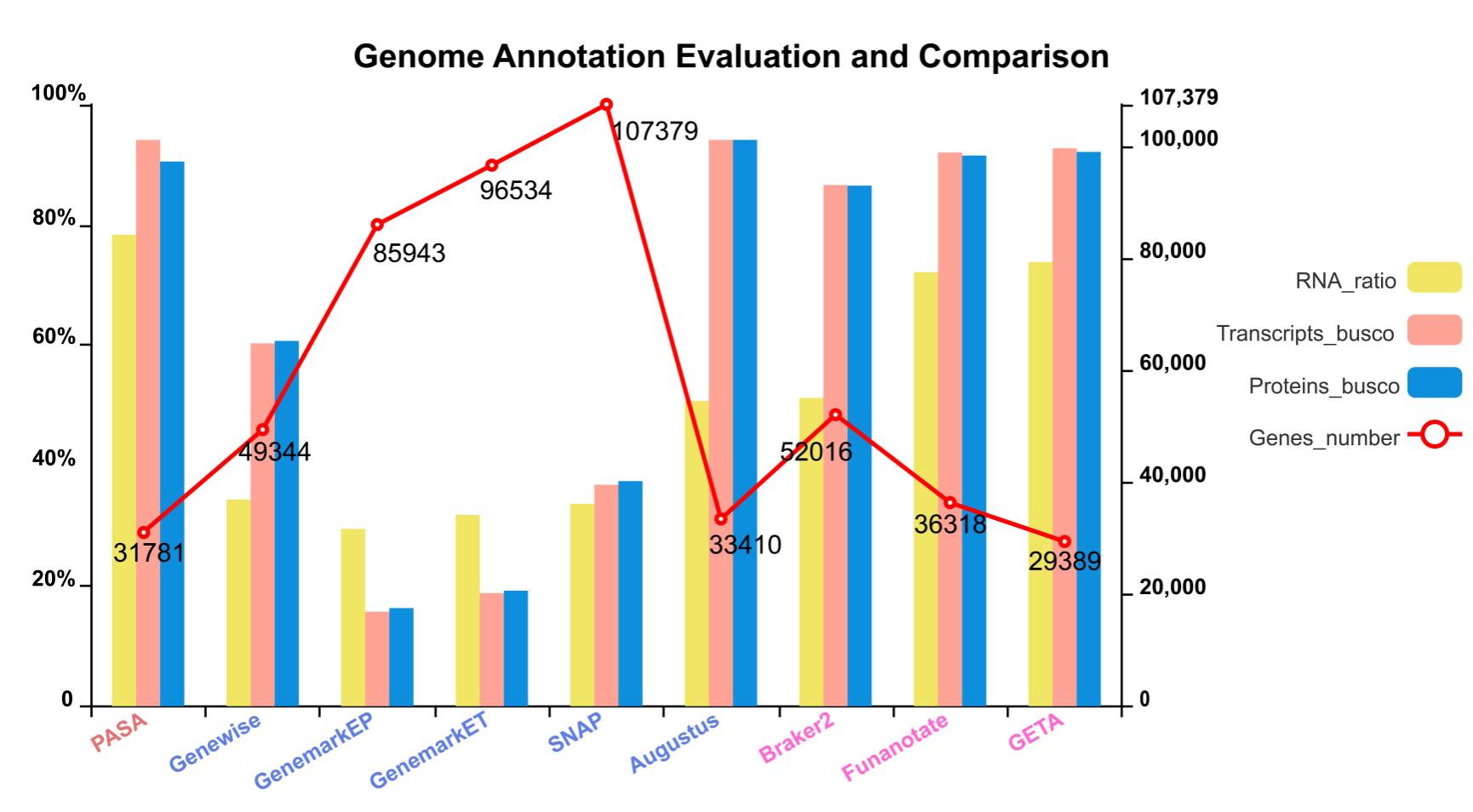
**

**Supplementary Figure S5.** **Results of different annotation software for *P. cordata.***

PASA was used for transcript-based gene prediction; GeneWise was used for homologous gene prediction; Augustus, GeneMark-ES/ET, and SNAP were used for *de novo* gene prediction; Braker2, Funanotate and GETA integrated the results of the three strategies. We evaluated the prediction results, including: the number of genes, the alignment rate of the transcriptome, and the completeness of the prediction of transcripts and proteins. Among the different software for gene prediction, GETA performed well in the completeness of gene prediction and the transcriptome alignment rate (Supplementary Figure 5, Supplementary Table S3). The GETA identified 29,389 protein-coding genes with an average length of 4,691  bp. The proportion of BUSCO genes that were completely or partially covered was 92.1% (Table 1).


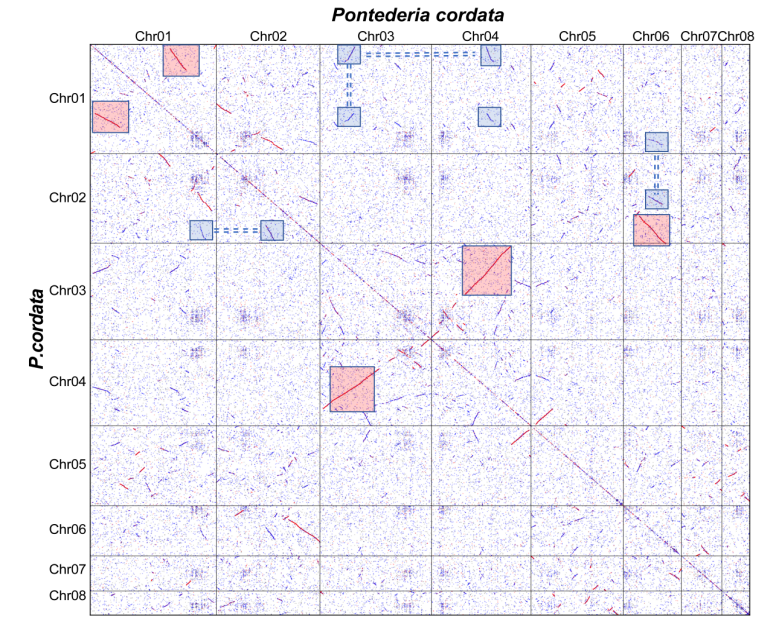

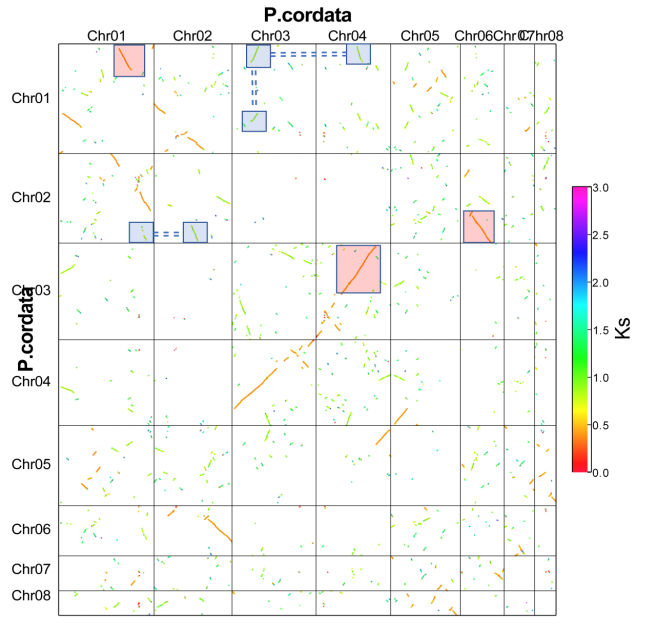

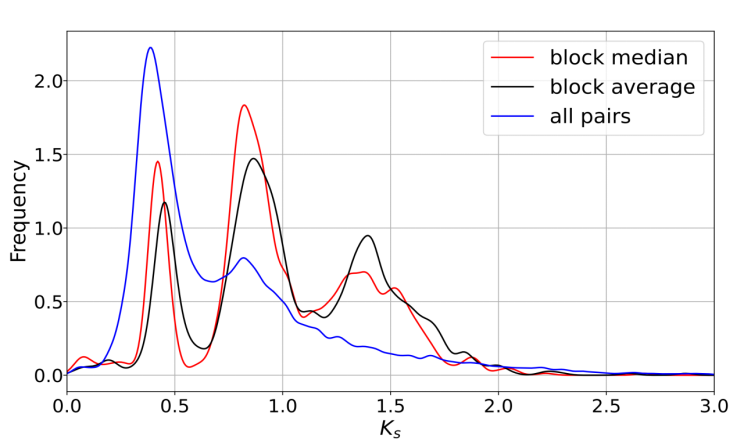

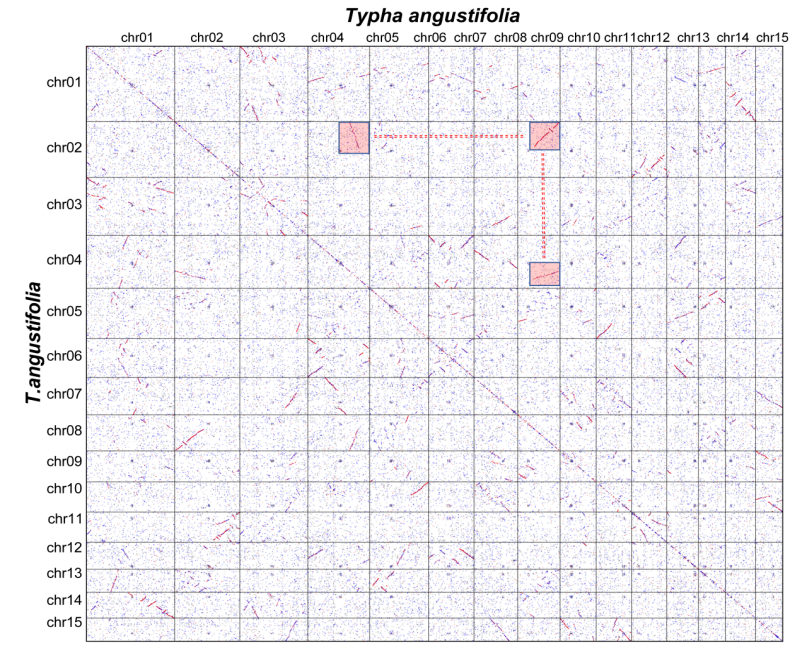

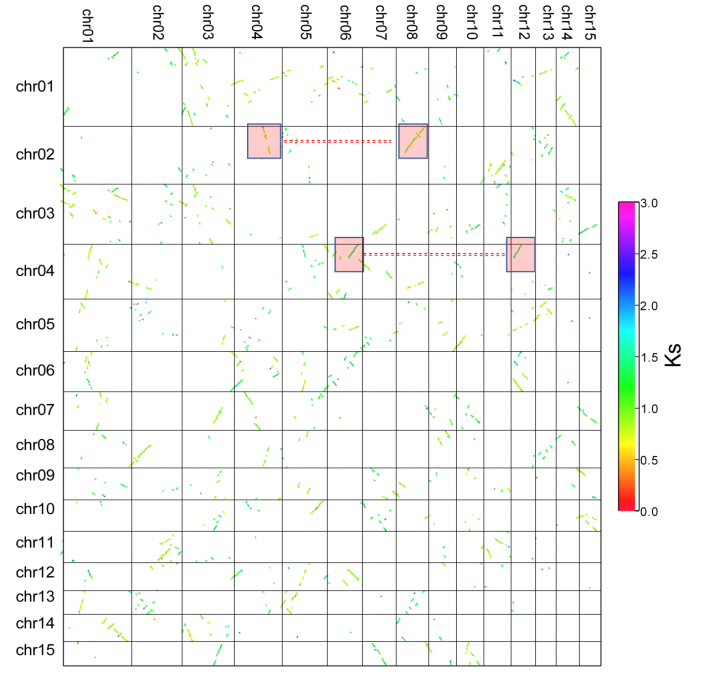

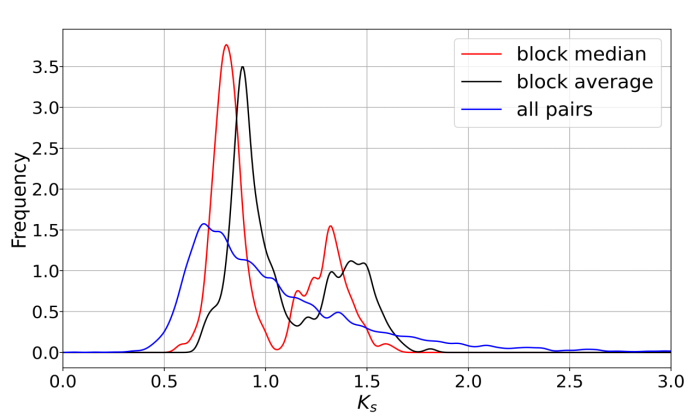

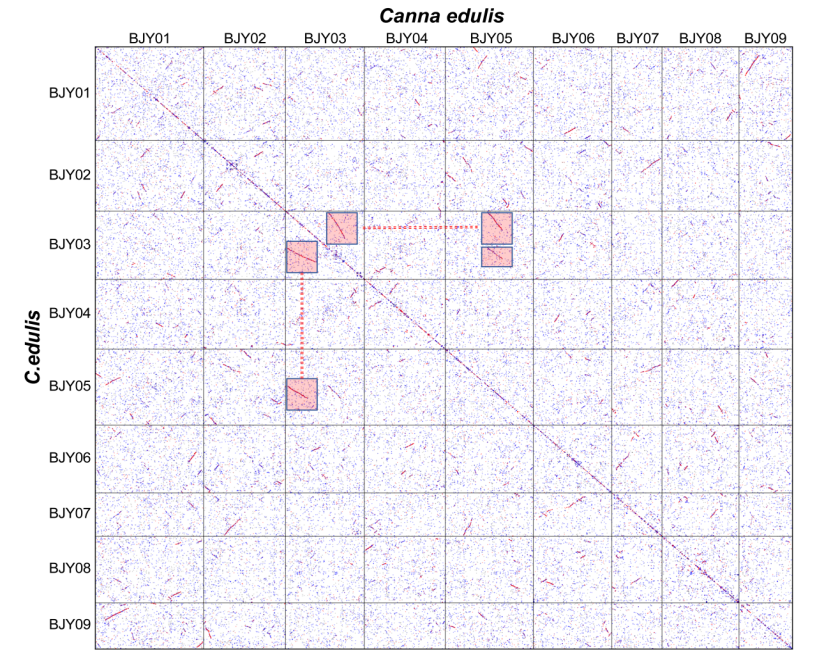

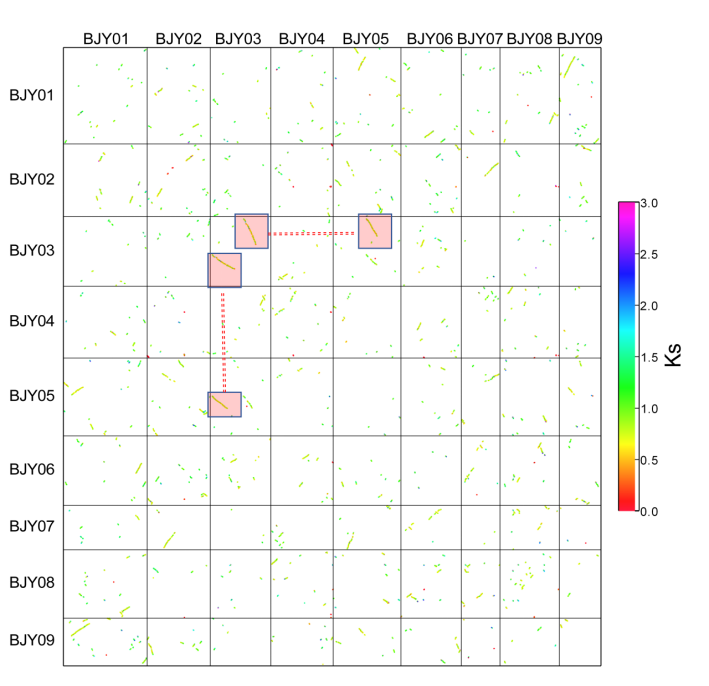

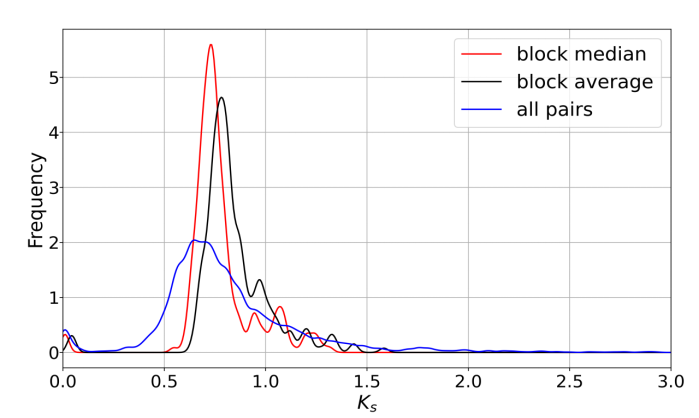


S6-a1

S6-b1

S6-c1

b3

b2

a3

a2

c2

c3

**Supplementary Figure S6. Homologous dot plots within each genome.**

The red dots represent their best-matched genes, the blue dots represent second best-matched ones, and the gray dots represent more ancient or dispersal duplicated genes. The regions surrounded by red frames were likely produced by **recent** WGD; those surrounded by blue frames were likely produced by **ancient** WGD. **a1-c1**: homologous gene dotplotting within each of ***P. cordata* ,** *Typha angustifolia*, and *Canna edulis*; a2-c2 : show the *Ks* of blocks in a dot plot; a3-c3 : *Ks* peaks in each genome.

S7-b


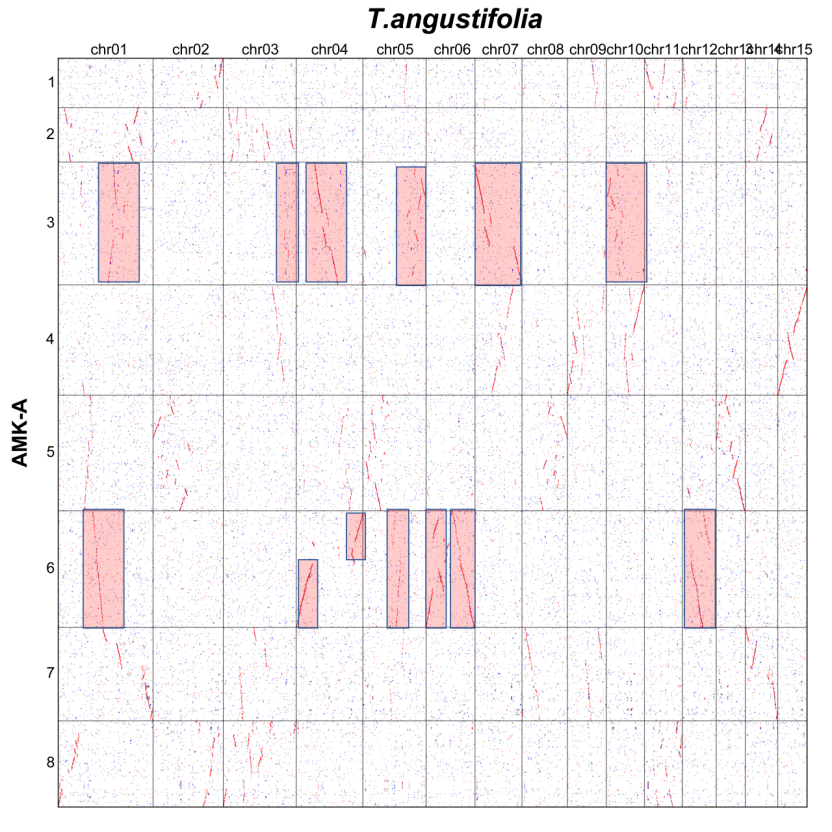

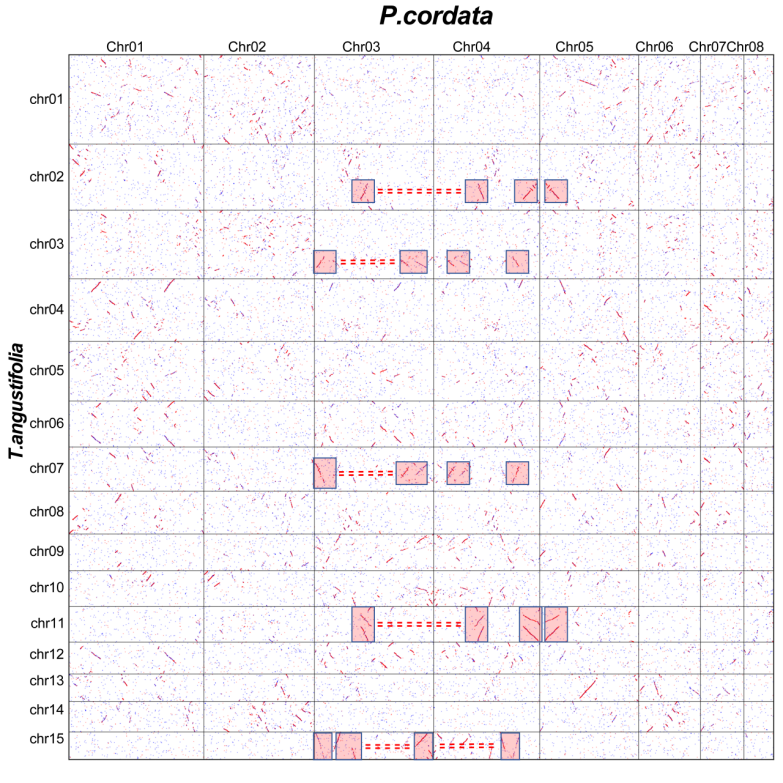

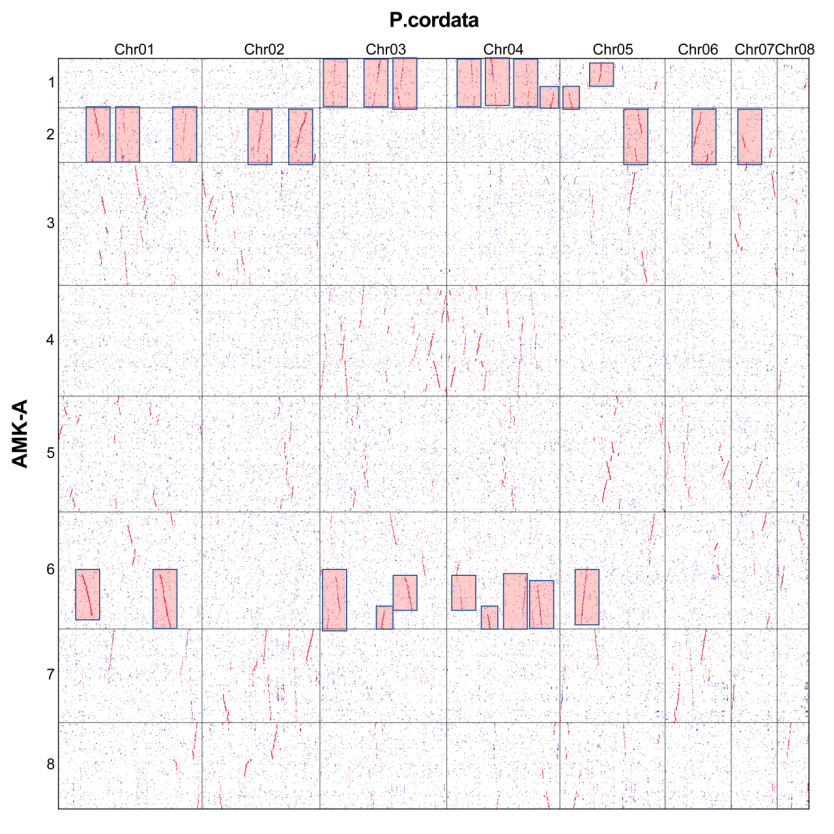


S7-c


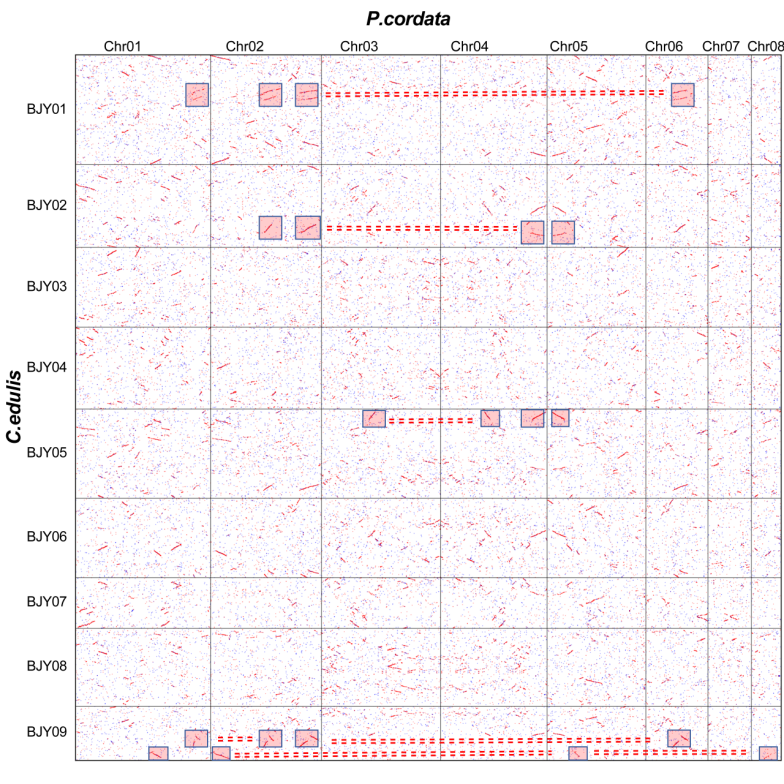


S7-a

S7-d

**Supplementary Figure S7.** **Homologous gene dotploting between *P. cordata* and the other commelinids genomes and the ancestral monocot karyotype (AMK-A).**

The red dots represent their best-matched genes, the blue dots represent second best-matched ones, and the gray dots represent ancient or dispersal homologs. The regions surrounded by red frames show the possible orthologous correspondence. The correspondence between chromosomes or chromosome regions between two plants was likely produced by WGD. **a-c**. Homolgous gene dot plots of **AMK-A**, *Typha angustifolia* (Poales), and *Canna edulis* (Zingiberales) with ***P. cordata* (Commelinaceae)**.

**Supplementary Figure S8. The gene duplication modes and numbers in *P. cordata.***

We identified 24,536 duplicated genes from the genome of *P. cordata*. The duplication modes of these genes were classified into five categories: 13,330 whole-genome duplicates (WGD, 45.36%), 1,777 tandem duplicates (TD, 6.05%), 874 proximal duplicates (PD, 2.97%), 5,995 transposed duplicates (TRD, 20.40%), and 2560 dispersed duplicates (DSD, 8.71%), and 853 other single genes.


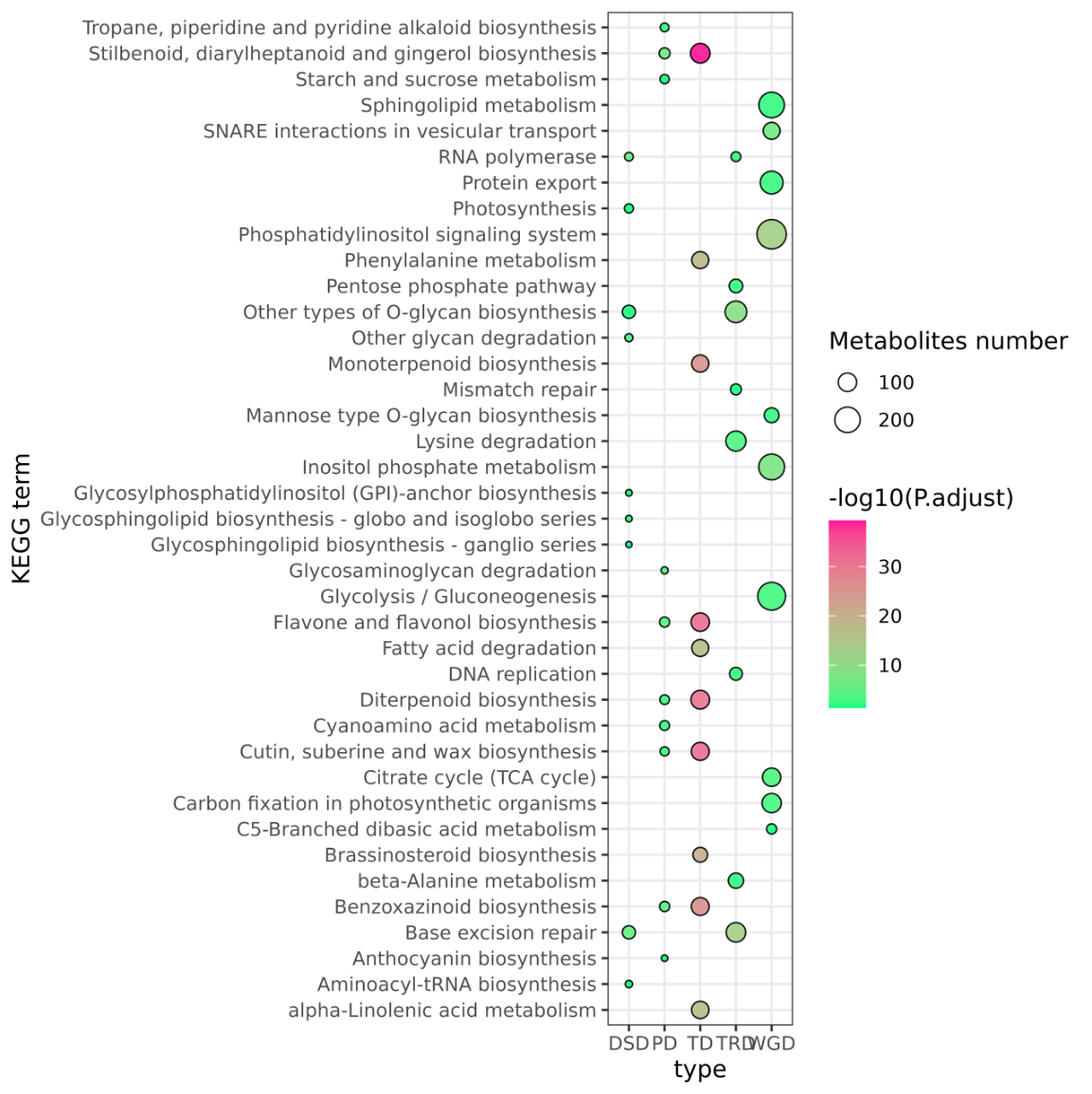

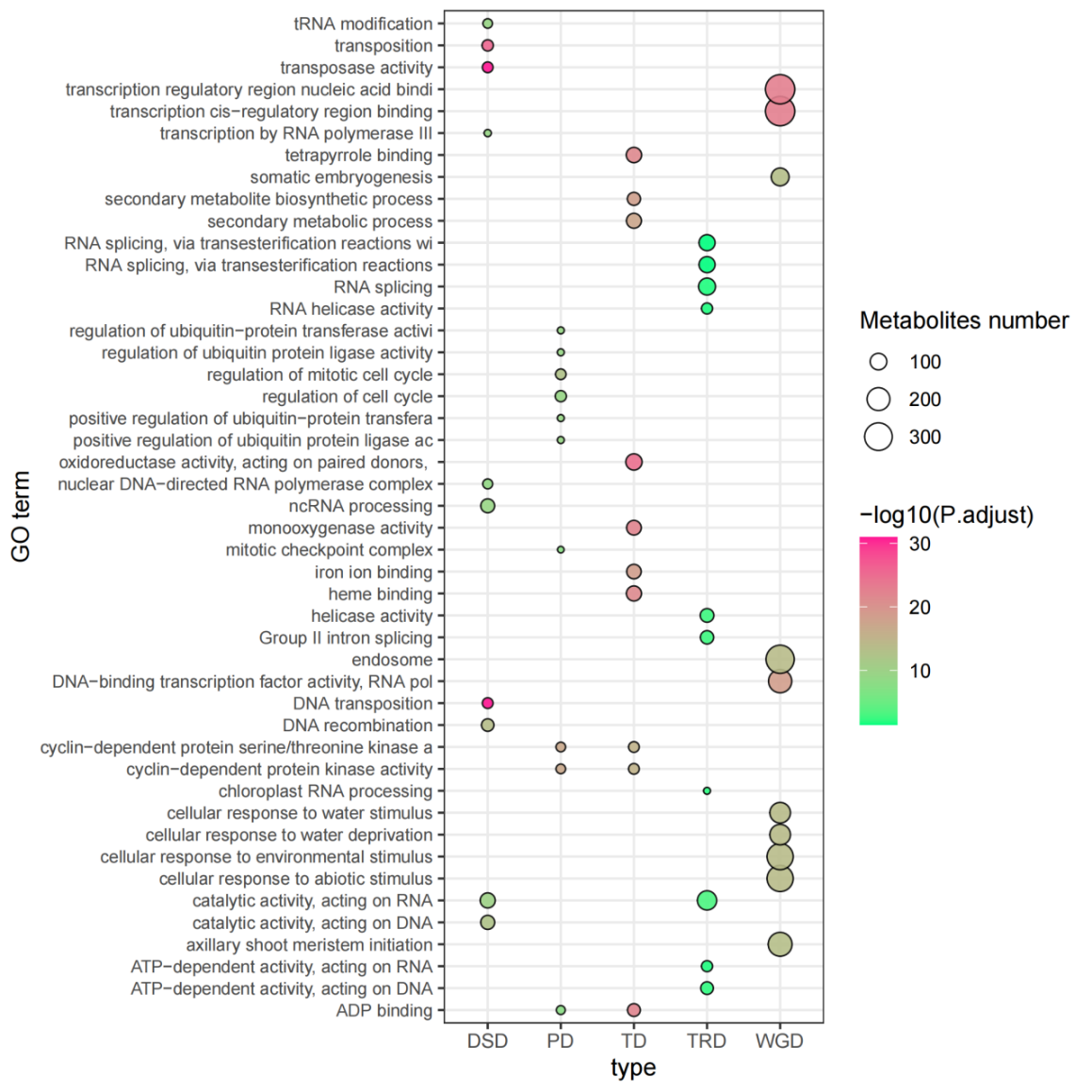


**Supplementary Figure S9. The GO and KEGG enrichment analyses of duplicated genes in *P. cordata.***


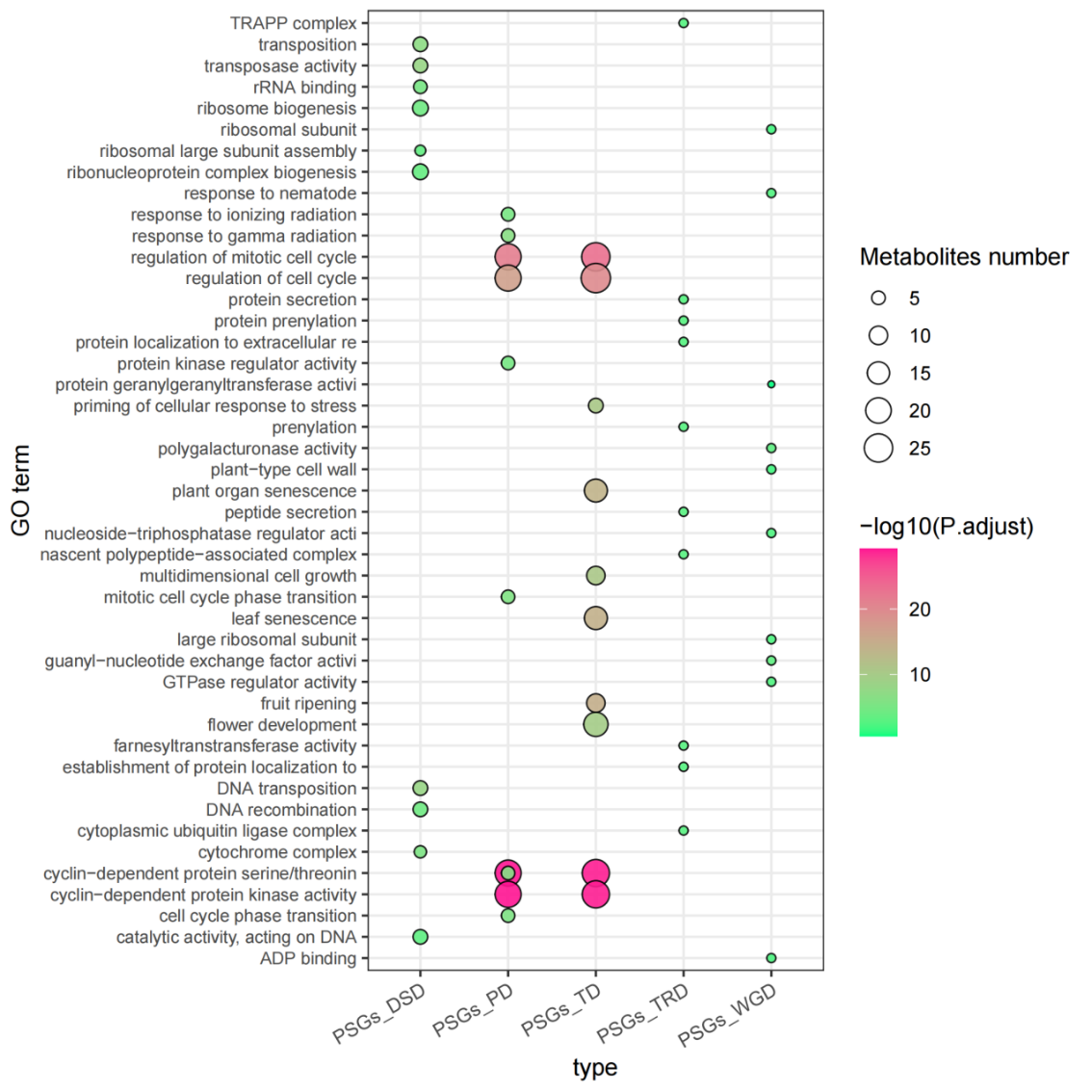

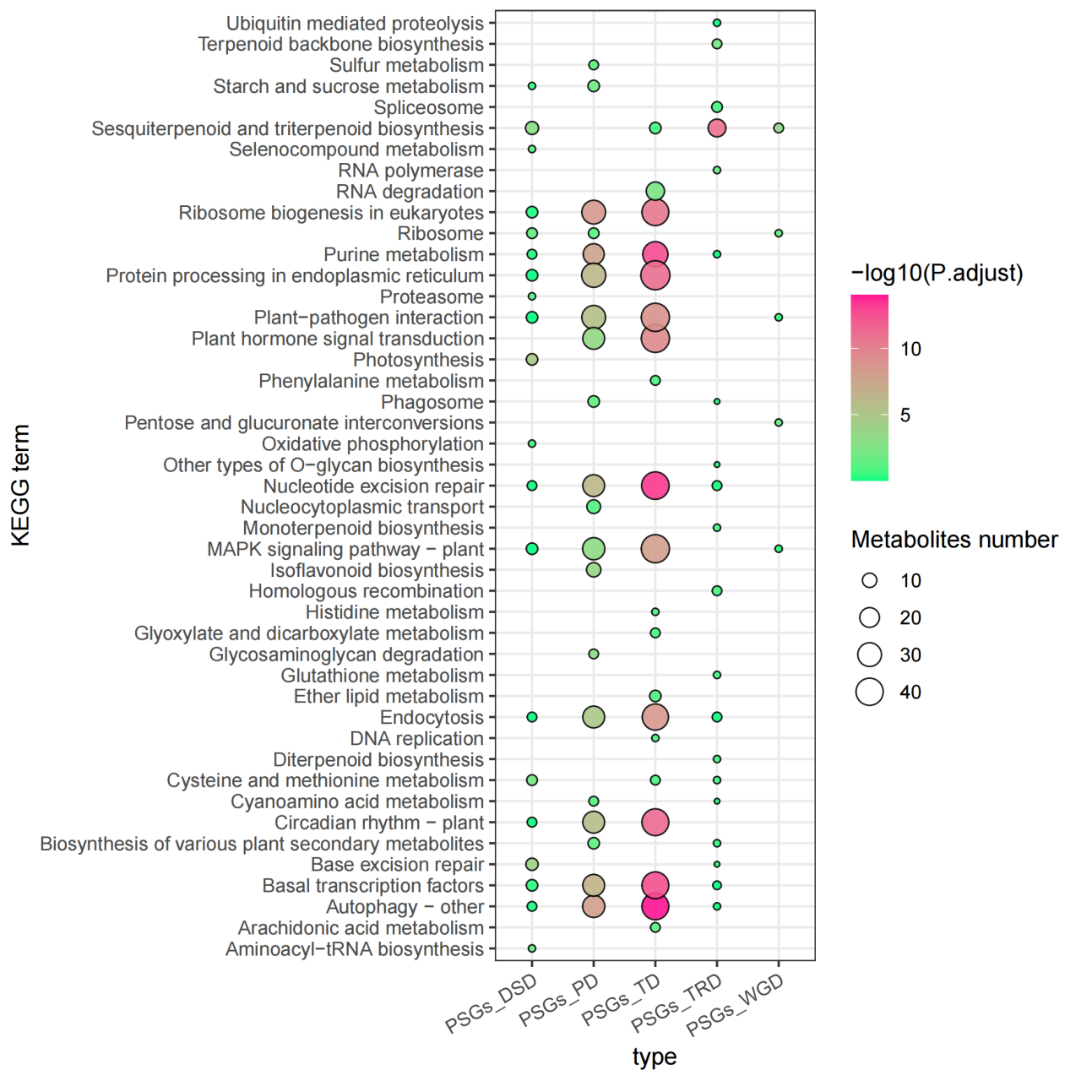


**Supplementary Figure S10.** **The GO and KEGG enrichment analyses of positively selected genes with different duplicated modes in *P. cordata.***


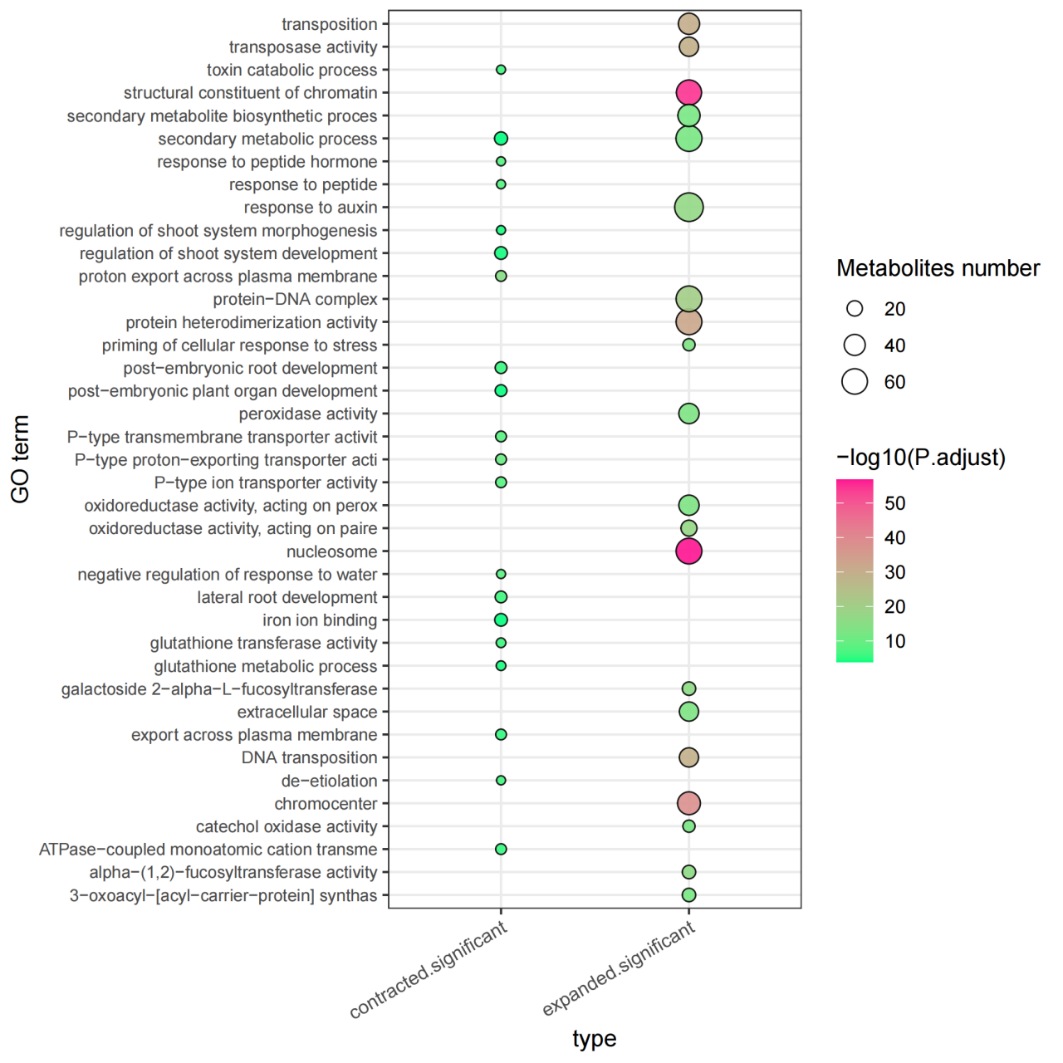

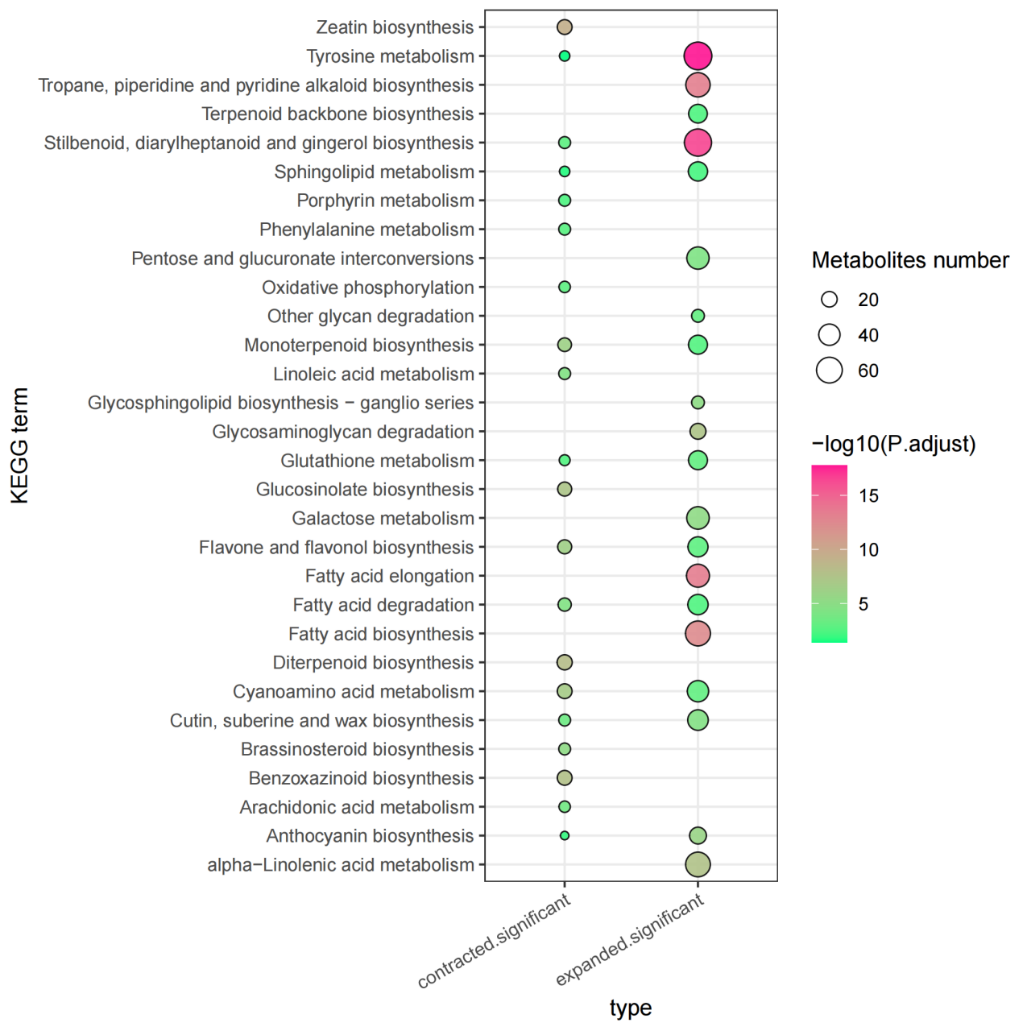


**Supplementary Figure S11.** **The GO and KEGG enrichment analyses of significantly contracted and expanded genes in *P. cordata.***


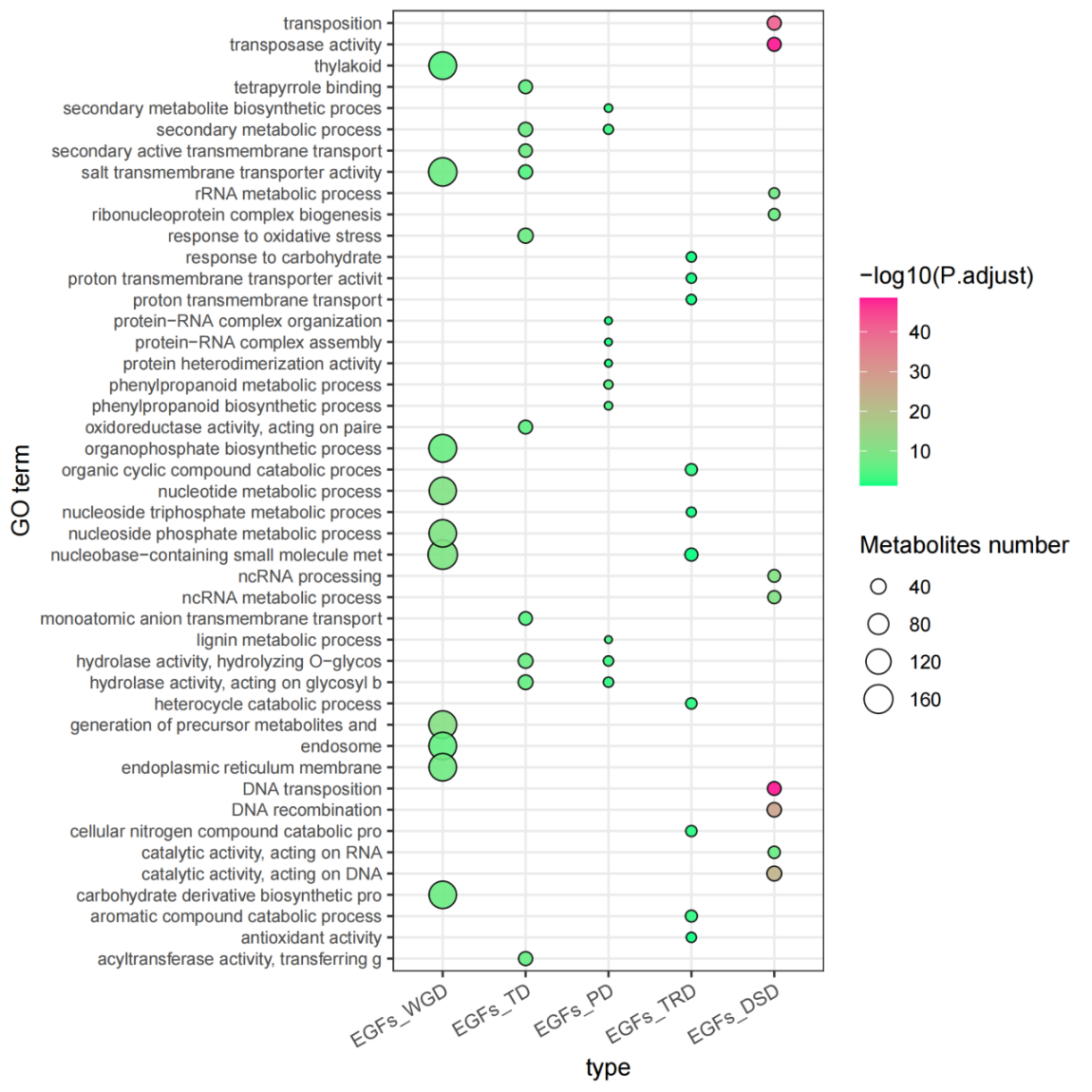

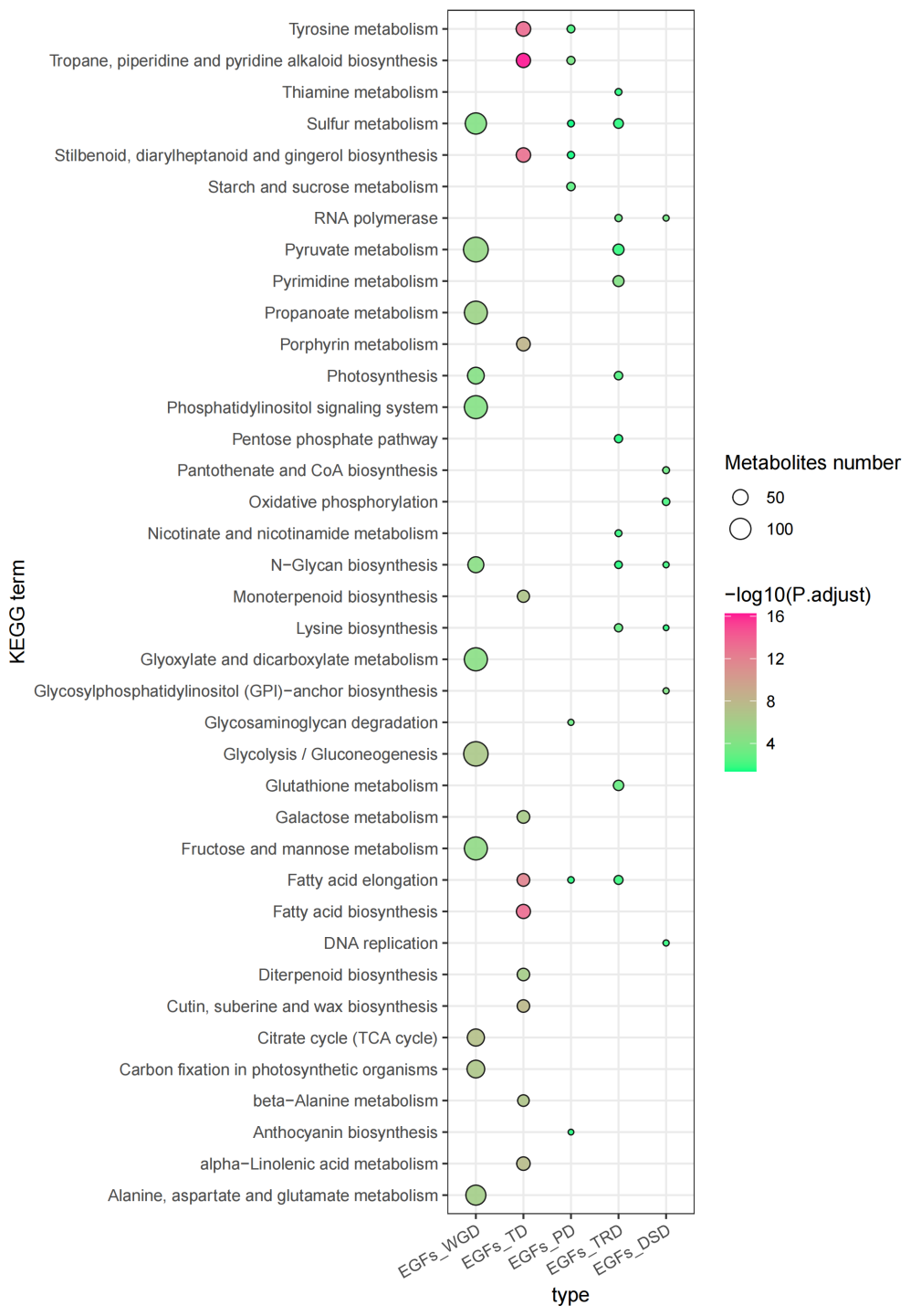


**Supplementary Figure S12.** **The GO and KEGG enrichment analysis of significantly contracted and expanded genes with different duplicated modes in *P. cordata.***

**Supplementary Figure S13. The phylogenetic trees of candidate genes in anthocyanin biosynthesis pathway in *P. cordata.***


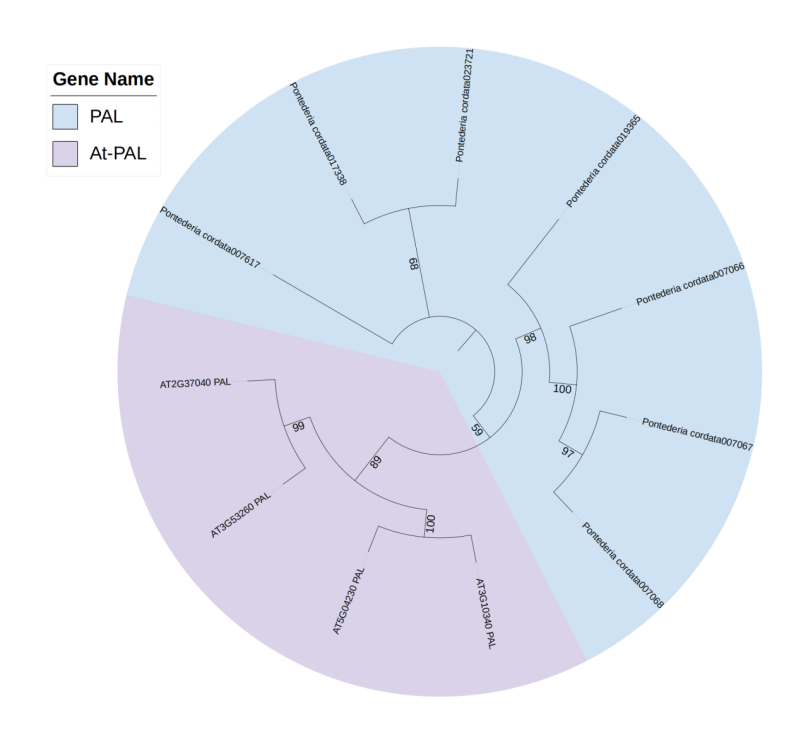

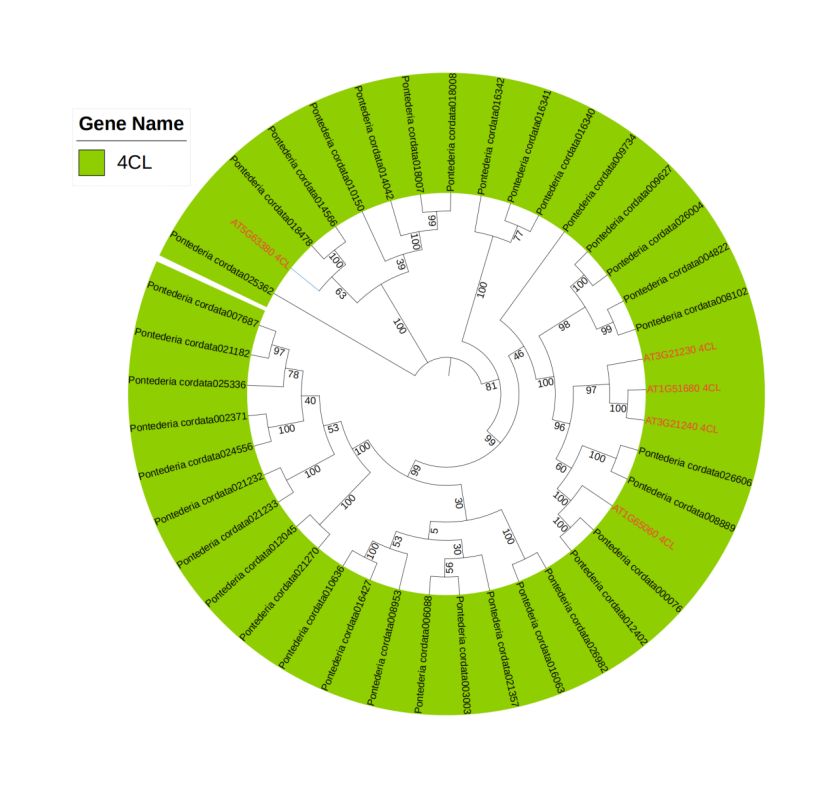

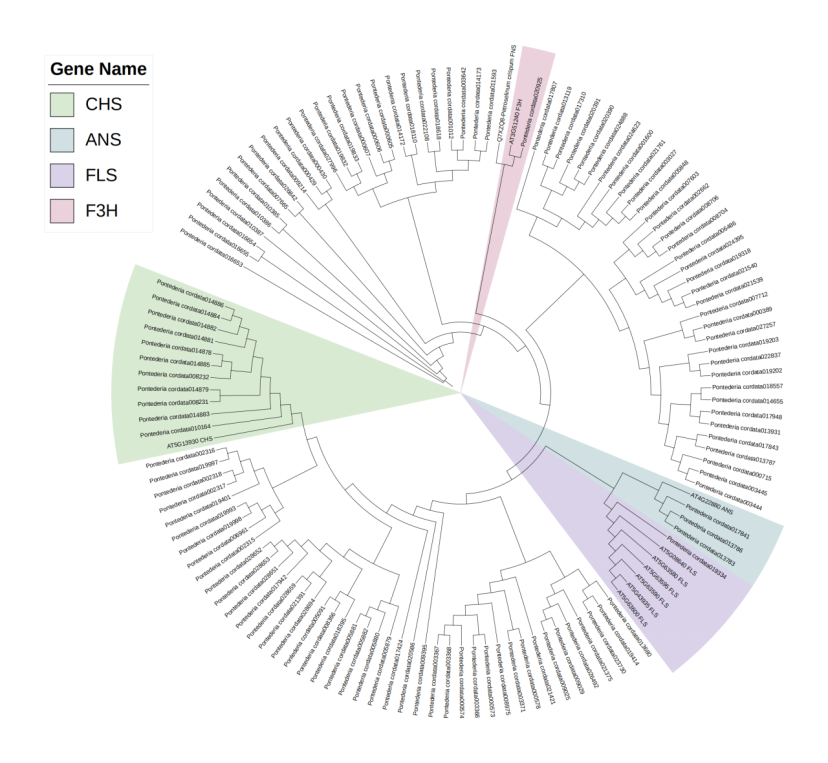

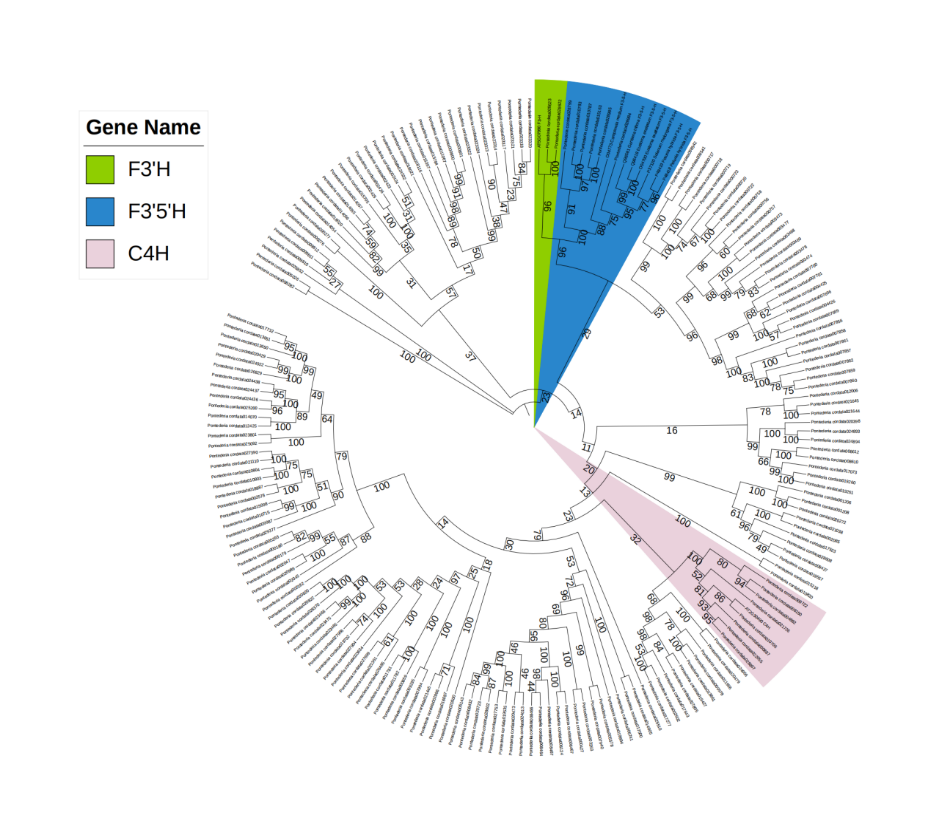

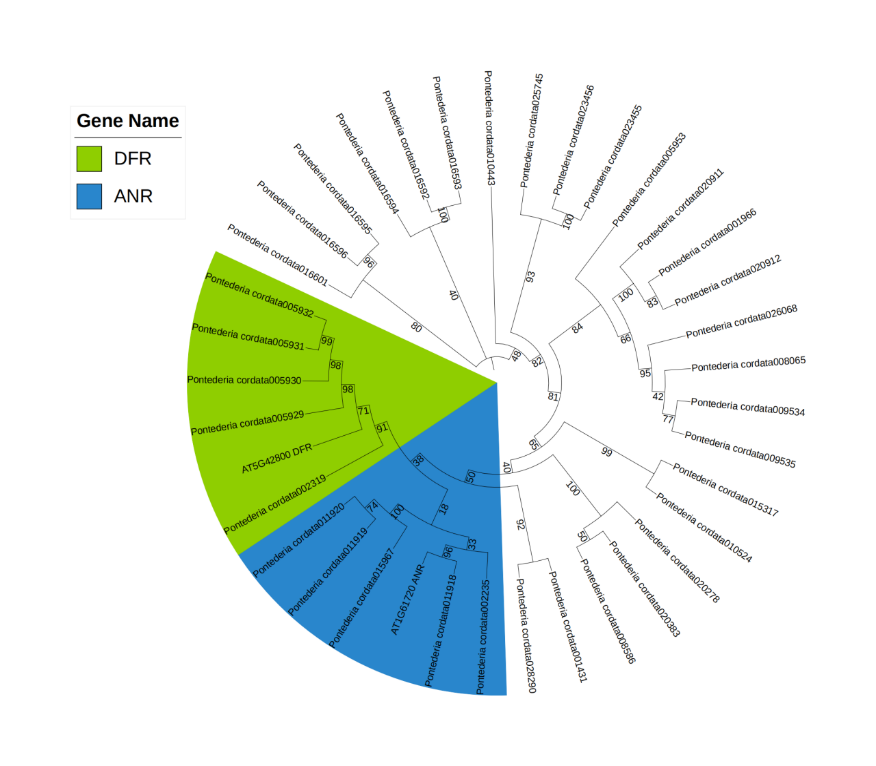

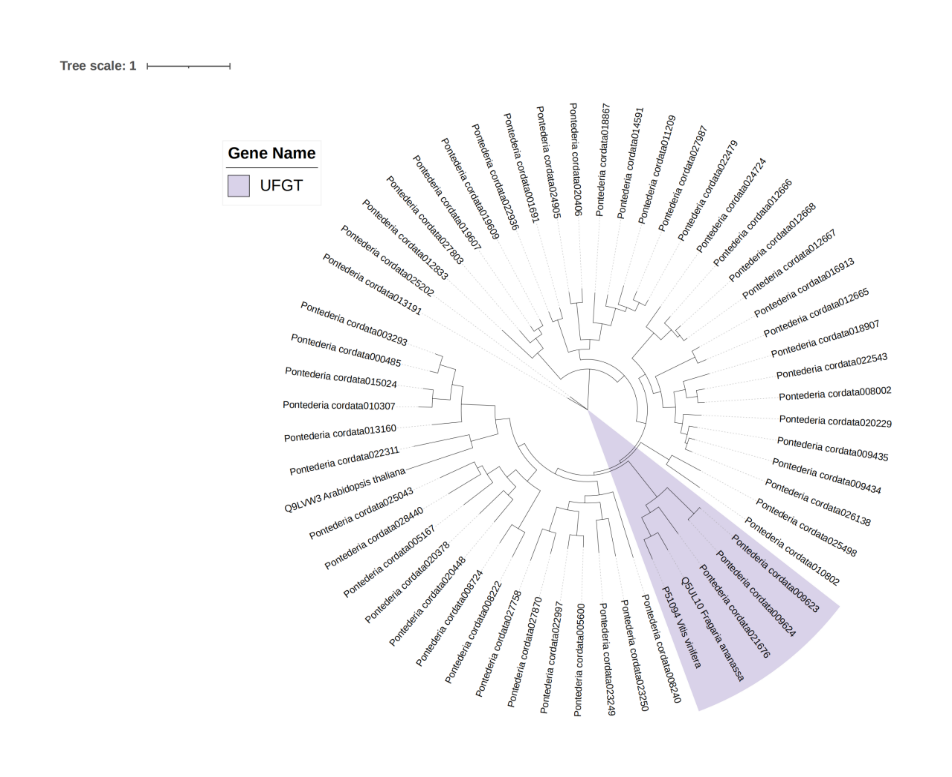


We determined the candidate genes through phylogenetic methods for the initially screened genes. The nodes highlighted in different colors include the final candidate genes.


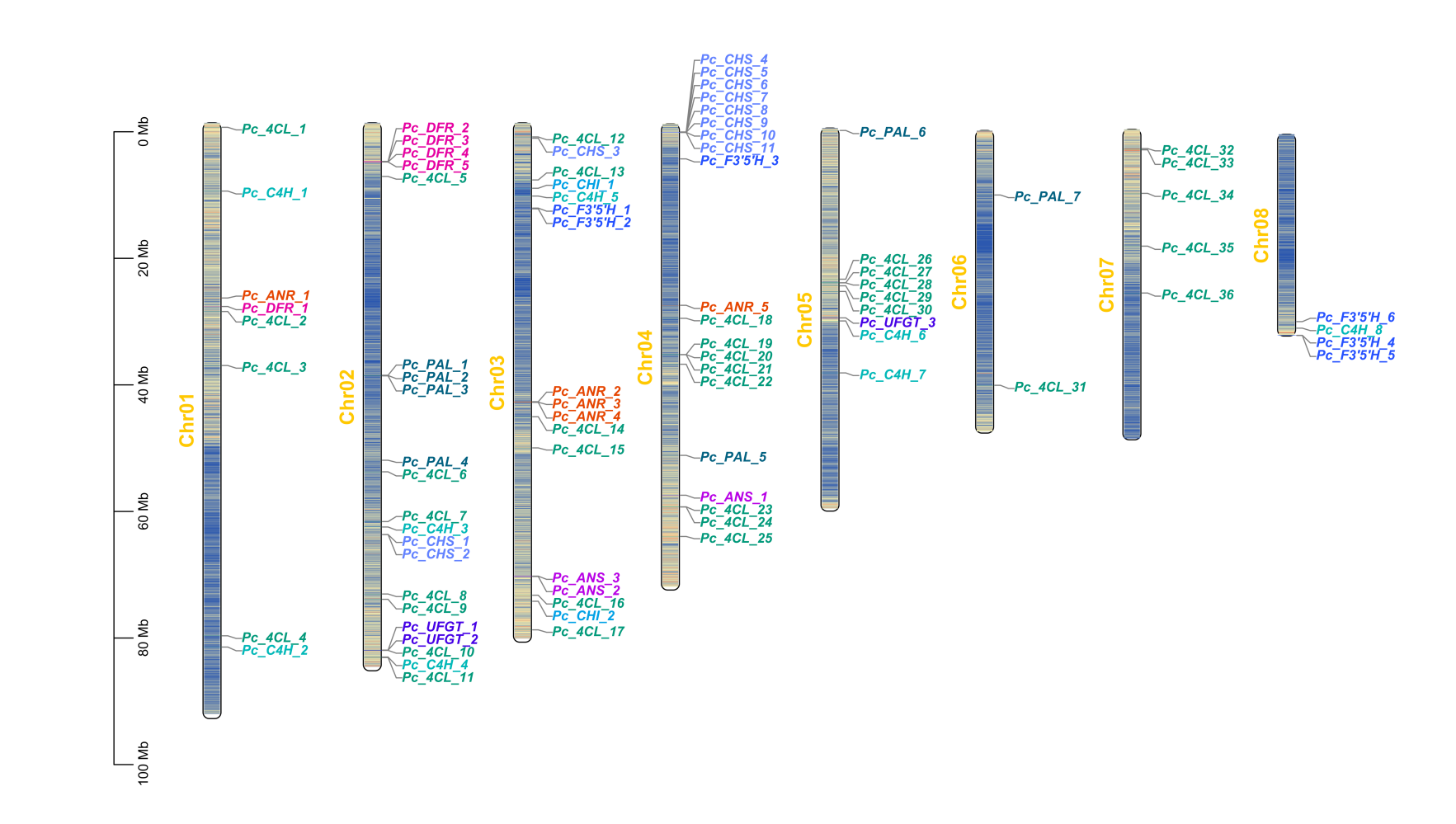


**Supplementary Figure S14. The distribution of genes involved in anthocyanin biosynthesis in *P. cordata*’s chromosomes.**

We identified 82 genes associated with anthocyanin synthesis in *P. cordata* (Supplementary [Table S](https://oup.silverchair-cdn.com/oup/backfile/Content_public/Journal/dnaresearch/30/4/10.1093_dnares_dsad012/1/dsad012_suppl_supplementary_tables.xlsx?Expires=1702112381&Signature=qLY8Z00su3w6zwiDwee--m33jILU1MjpzjV9143n8XIxLVYwkm7SHJVdbJ1Ns9j2~diFW4irtELgfXSkJJ9Cw5keBdta9E~qGO~Wfa6R764q-pkZGCU9EK1CYM87QluFnS0urWTJj3shj2V2gSga6NNTqLGRFRxAKrDURKcd-WuHdPN4UOVNFfk7MeQTQebI4BHfXvDGMYFJ-N-VKIOHz6V1OzEwDjDkg8IgRhVeWzrq1Hagwej~d5IHy8b~XyHux-aX39F3c6aGyS-zAVIN8I4r0meCBdhbVLvW7JmljPvh-gYRfnPbvFHrX1MrhQADABveJp1FdWgBBVC3MkmWeg__&Key-Pair-Id=APKAIE5G5CRDK6RD3PGA)10), including *PAL* (7), *C4H* (8), *4CL* (36), *CHS* (11), *CHI* (2), *F3H* (1), *F3*ʹ*5*ʹ*H* (6), *DFR* (5), *ANS* (3), *UFGT* (3).

Theses genes are distributed on eight chromosomes (Supplementary Figure14), and the number of genes distributed on chromosome 2 (Chr02, 21) and 3 (Chr03, 17) is the largest, and chromosome 6 (Chr06, 2) is the least. These genes are divided into different duplication types, of which WGD (40%) and TD (23%) are the main duplication types of most genes (Supplementary [Table S](https://oup.silverchair-cdn.com/oup/backfile/Content_public/Journal/dnaresearch/30/4/10.1093_dnares_dsad012/1/dsad012_suppl_supplementary_tables.xlsx?Expires=1702112381&Signature=qLY8Z00su3w6zwiDwee--m33jILU1MjpzjV9143n8XIxLVYwkm7SHJVdbJ1Ns9j2~diFW4irtELgfXSkJJ9Cw5keBdta9E~qGO~Wfa6R764q-pkZGCU9EK1CYM87QluFnS0urWTJj3shj2V2gSga6NNTqLGRFRxAKrDURKcd-WuHdPN4UOVNFfk7MeQTQebI4BHfXvDGMYFJ-N-VKIOHz6V1OzEwDjDkg8IgRhVeWzrq1Hagwej~d5IHy8b~XyHux-aX39F3c6aGyS-zAVIN8I4r0meCBdhbVLvW7JmljPvh-gYRfnPbvFHrX1MrhQADABveJp1FdWgBBVC3MkmWeg__&Key-Pair-Id=APKAIE5G5CRDK6RD3PGA)10). For example, all repeated copies of *C4H* are caused by WGD, and 8 of 11 *CHS* genes are caused by TD. Subcellular Localization analysis showed that most genes are expressed in chloroplast, cytoplasm and plasma membrane. For example, F3ʹ5ʹH is expressed in chloroplasts.

**
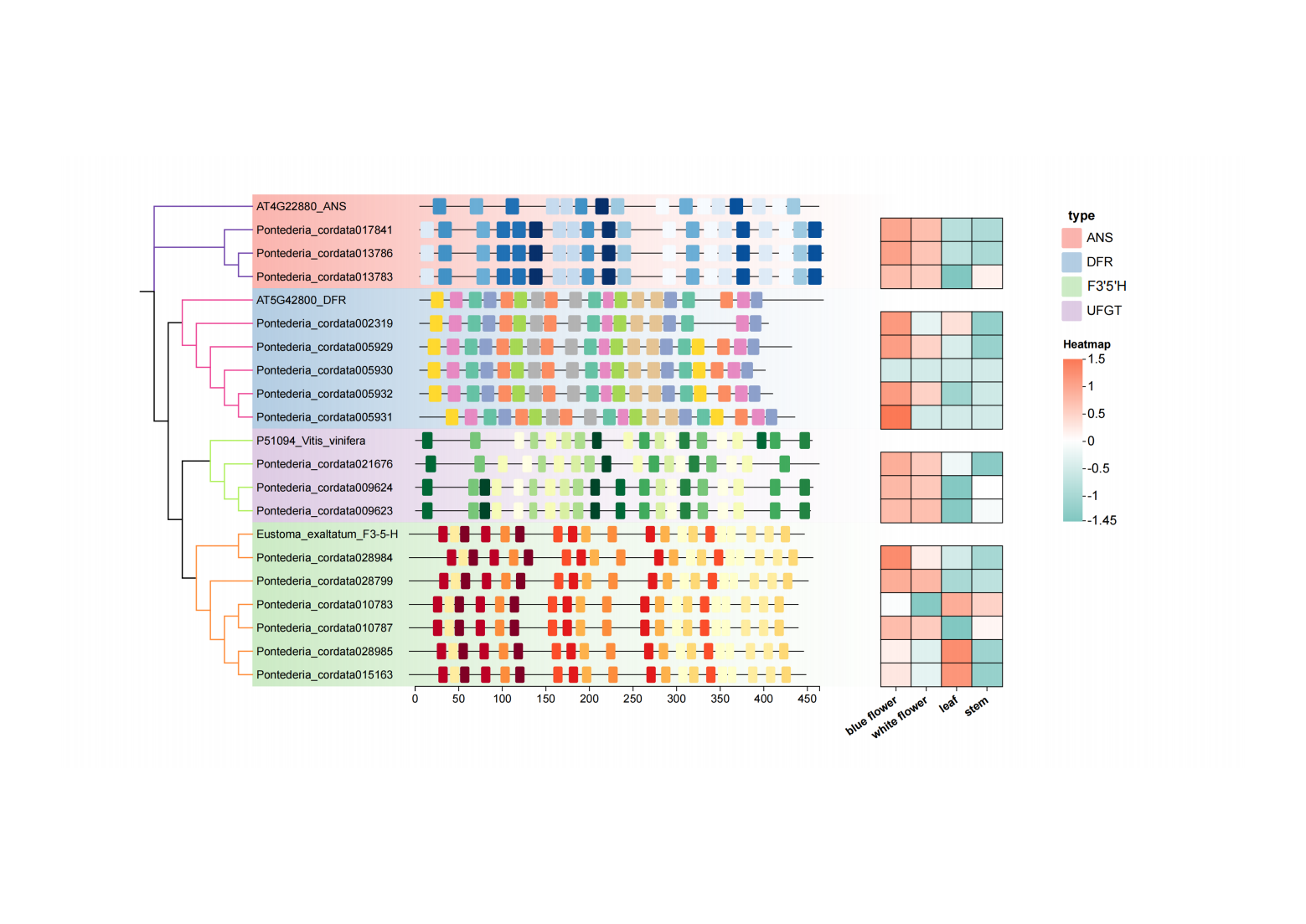
Supplementary Figure S15. The motif of anthocyanin biosynthesis candidate genes in *P. cordata.***

The physicochemical properties and conserved motifs of these genes were similar to those of reference genes (Supplementary [Table S](https://oup.silverchair-cdn.com/oup/backfile/Content_public/Journal/dnaresearch/30/4/10.1093_dnares_dsad012/1/dsad012_suppl_supplementary_tables.xlsx?Expires=1702112381&Signature=qLY8Z00su3w6zwiDwee--m33jILU1MjpzjV9143n8XIxLVYwkm7SHJVdbJ1Ns9j2~diFW4irtELgfXSkJJ9Cw5keBdta9E~qGO~Wfa6R764q-pkZGCU9EK1CYM87QluFnS0urWTJj3shj2V2gSga6NNTqLGRFRxAKrDURKcd-WuHdPN4UOVNFfk7MeQTQebI4BHfXvDGMYFJ-N-VKIOHz6V1OzEwDjDkg8IgRhVeWzrq1Hagwej~d5IHy8b~XyHux-aX39F3c6aGyS-zAVIN8I4r0meCBdhbVLvW7JmljPvh-gYRfnPbvFHrX1MrhQADABveJp1FdWgBBVC3MkmWeg__&Key-Pair-Id=APKAIE5G5CRDK6RD3PGA)11, Supplementary Figure 15), which also showed the reliability of these candidate genes.

We found that the downstream biosynthetic genes, *F3*ʹ*5*ʹ*H*, *DFR*, *ANS*, and *UFGT*, are highly expressed in violet flowers, compared with white flowers, leaf and stem tissues. The high expression of these genes in violet flowers leads to the accumulation of anthocyanins, resulting in their characteristic purple appearance. In contrast, the *DFR* and *UFGT* genes in white flowers are virtually unexpressed.


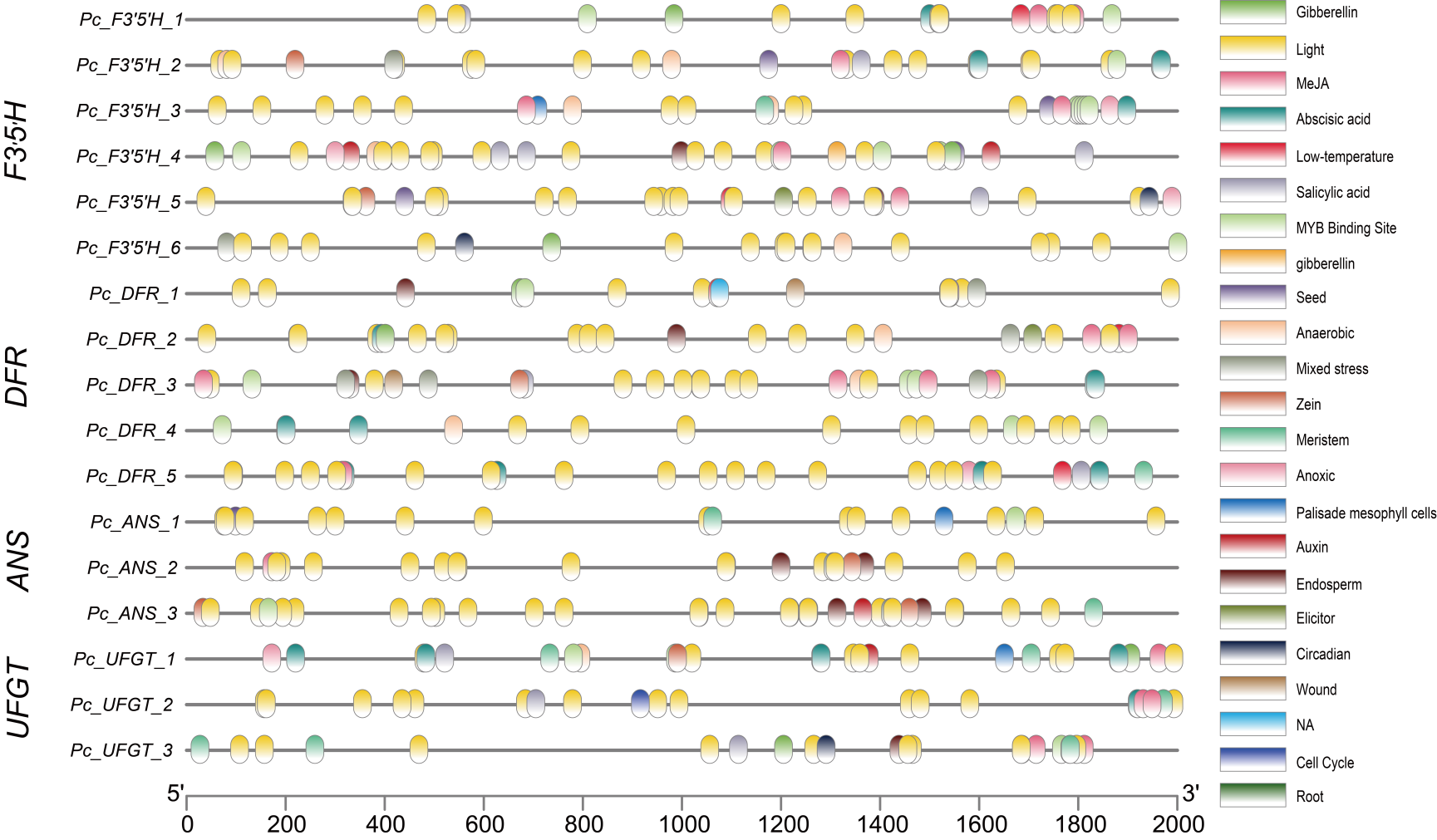


**Supplementary Figure S16. Prediction and analysis of cis-acting elements in the *F3ʹ5ʹH*, *DFR*, *ANS* and *UFGT* gene family promoter regions.**

The distribution of cis-acting elements in the *F3ʹ5ʹH*, *DFR*, *ANS*, and *UFGT* promoter regions (−2000 bp). Different colors and shapes represent different types of cis-acting elements. The ruler at the bottom indicates the direction and length of the promoter sequence.


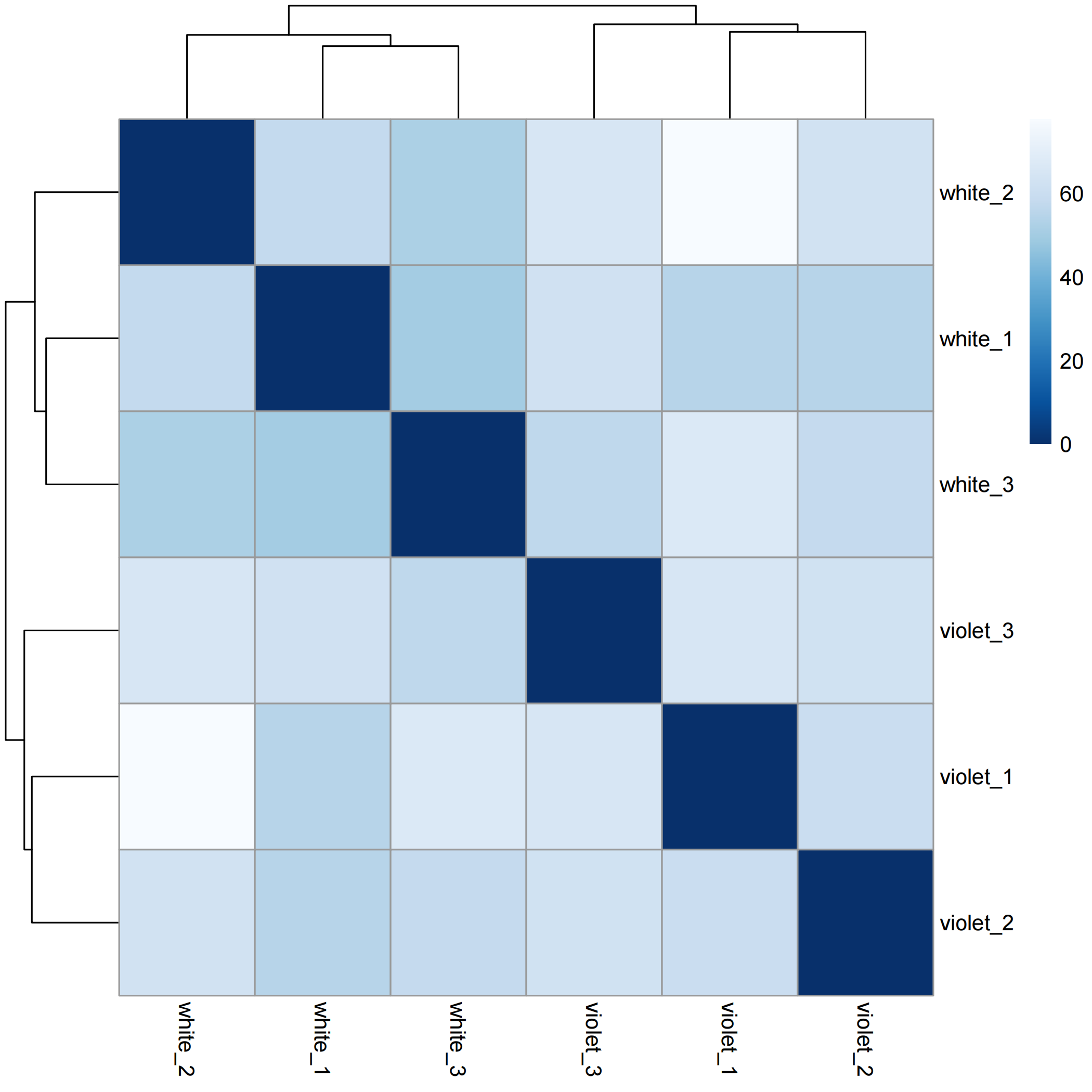

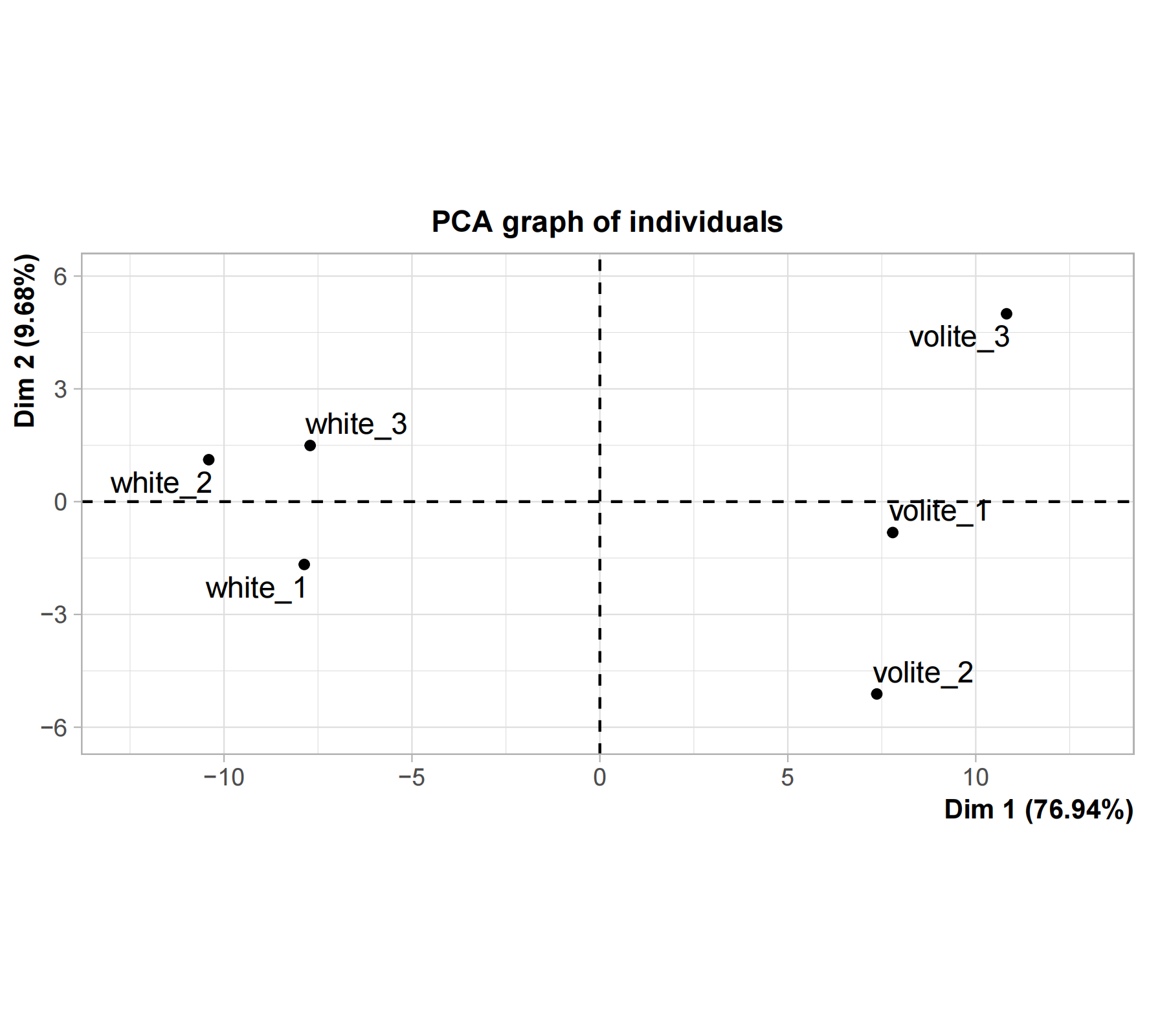

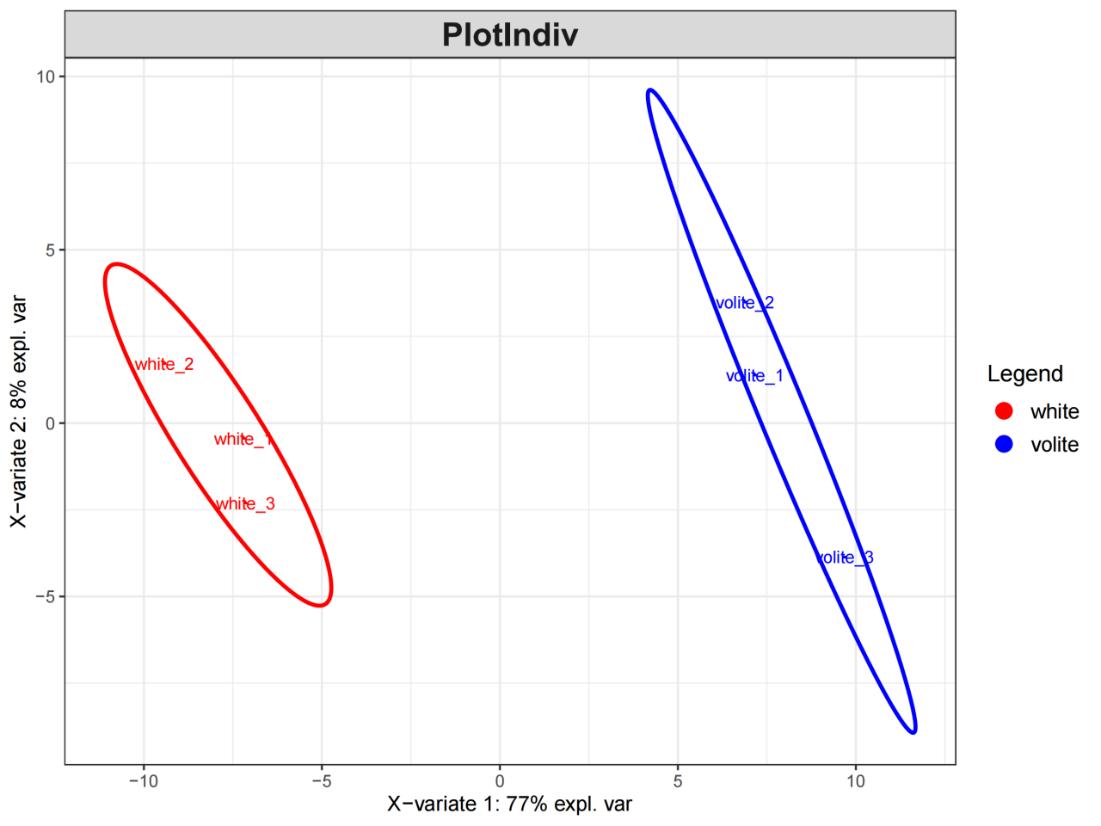


**Fig** S17-a

1

**Fig** S17-c

**Fig** S17-b

**Supplementary Figure S17. Sample clustering analysis.**

To verify the consistency and reliability of samples, we performed PCA, hierarchical clustering analysis, and PLS-DA analysis on the six transcriptomes generated in this study. Although the six transcriptomes are derived from different individuals, the samples were separated into two groups by flower color, which supported the consistency of our data. **Fig S17-a**: Partial least squares discriminant analysis (PLS-DA) method is used to cluster samples. **Fig S17-b**: Hierarchical Clustering Method (rld); **Fig S17-c**: Principal Component Analysis (PCA).

**Fig** S18-a

**Fig** S18-b


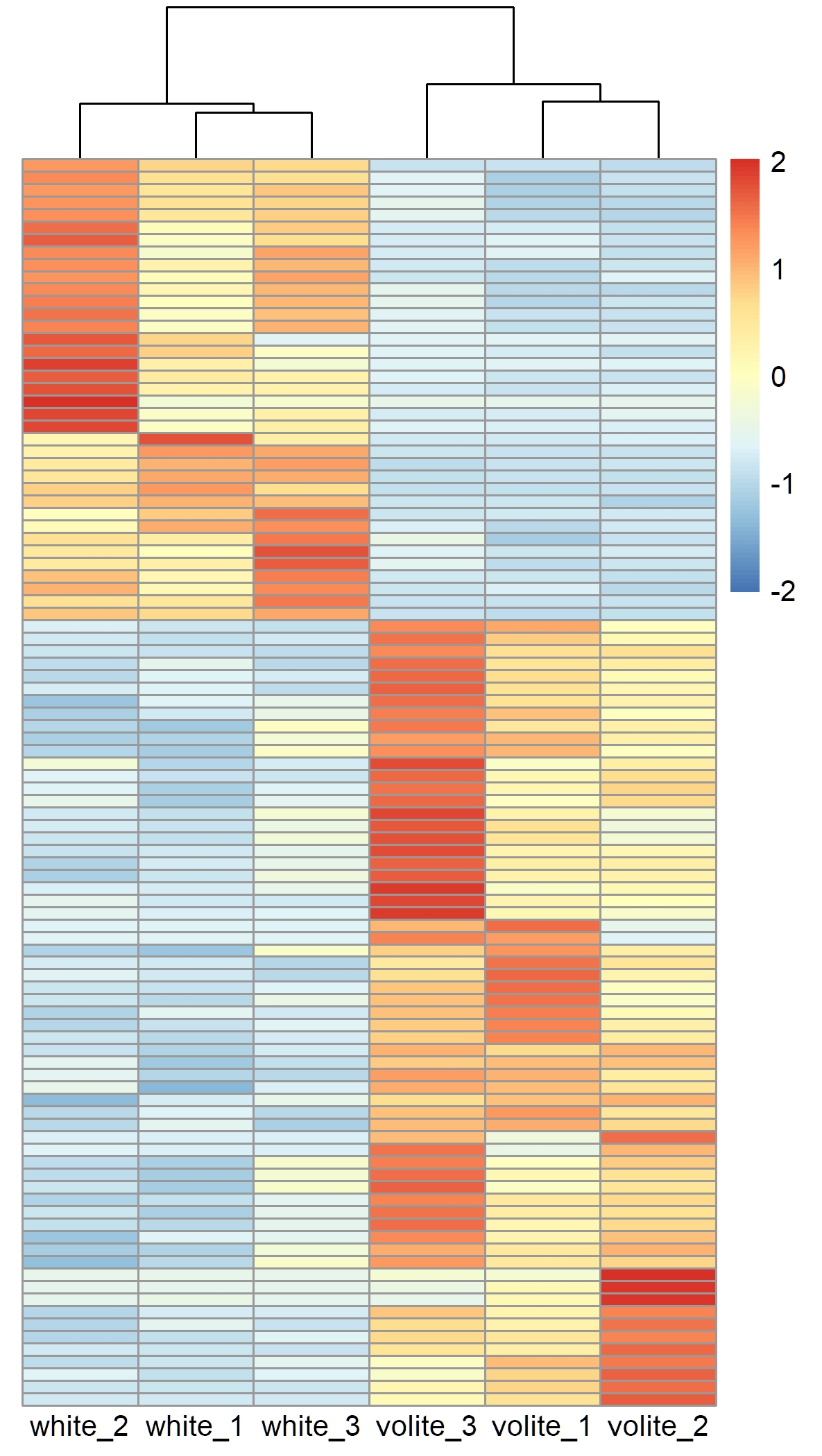

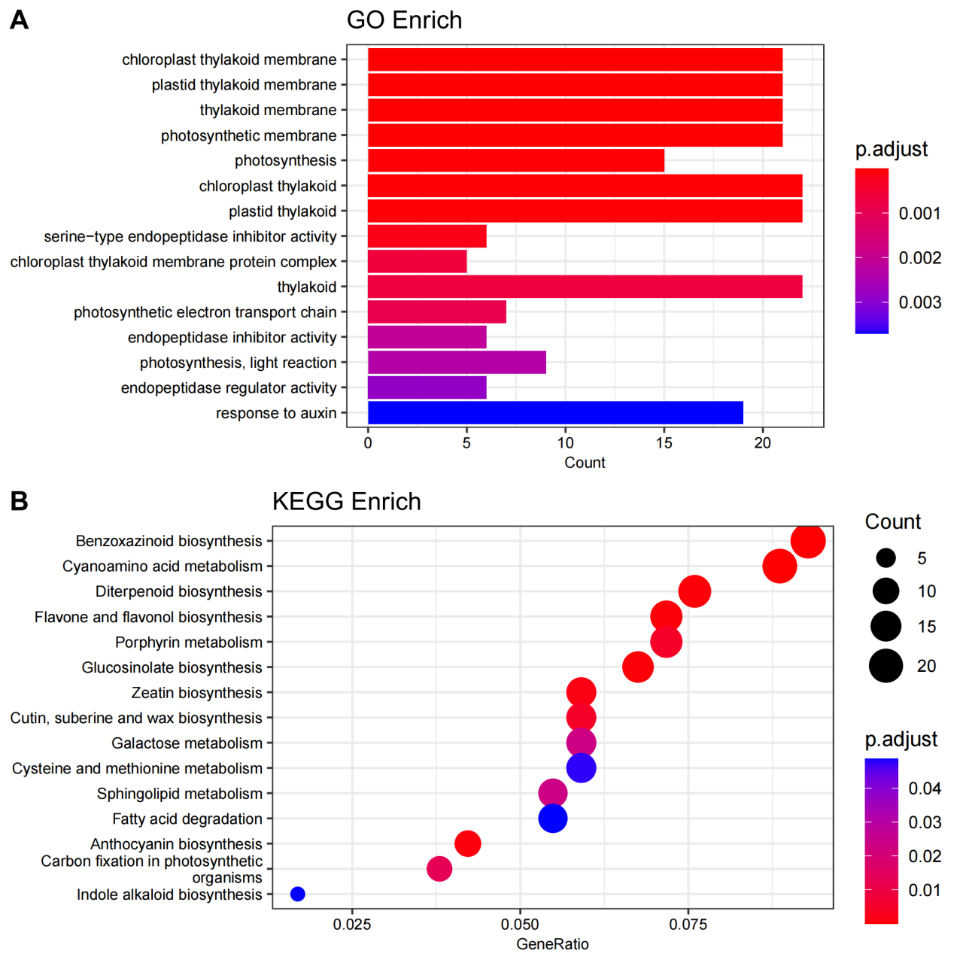


**Supplementary Figure S18. Differential gene expression analysis of violet and white flowers of *P. cordata*.**

**Fig S18-a**: Heatmap of the differential expressed genes in three violet and white flowers. The red represents high gene expression, the blue represents low gene expression. **Fig** **S18-b**: Functional enrichment analysis of upregulated genes in violet flowers, A: GO enrichment, B: KEGG enrichment, the color change represents the significance of the results, and the size of the dots represents the number of genes enriched under that entry.
